# P16-positive senescent cells promote DKD by the dysregulation of glycolysis and mitochondrial metabolism

**DOI:** 10.1038/s41420-025-02650-2

**Published:** 2025-07-30

**Authors:** Xiao Lu, Jiao Wu, Ewud Agborbesong, Xiaogang Li

**Affiliations:** 1 Department of Internal Medicine, Mayo Clinic, Rochester, MN 55905 USA; 2 Department of Nephrology, Fifth Hospital of Shanxi Medical University (Shanxi Provincial People’s Hospital), Taiyuan, China; 3 Biochemistry and Molecular Biology, Mayo Clinic, Rochester, MN 55905 USA

**Keywords:** Diabetes complications, Kidney diseases

## Abstract

Diabetic kidney disease (DKD) is characterized by kidney damage and abnormal renal energy metabolism, but the molecular mechanism of DKD is still unclear. In this study, we show that p16- positive senescent cells are an important regulator in the progression of DKD. The expression of p16 and senescence are increased in the kidneys of DM mice and DKD patients. To better understand the role of p16 in DKD, we induce type 1 diabetes in INK-ATTAC mice, a mouse model that allows the selective ablation of p16-expressing cells upon administration of the drug AP20187. We found that clearance of p16-positive cells, most of them are senescent cells, (1) decreased senescence and the expression of the components of the senescence-associated secretory phenotypes (SASPs), (2) restored kidney adenosine triphosphate (ATP) content, (3) decreased the expression of the key glycolytic genes to improve the metabolic reprogramming, (4) normalized the mitochondrial metabolism through AMPK and mTOR pathway, resulting in an amelioration of the progression of DKD. In addition, p16 mediated the blocking of the cell cycle is through the CDK4-Rb pathway in DKD kidneys. This study suggests that pharmacological deletion of p16-positive senescent cells may be a novel therapeutic strategy for DKD treatment.

## Introduction

Diabetic kidney disease (DKD), also called diabetic nephropathy, is a chronic kidney disease with serious complications of diabetes mellitus (DM), resulting in a high morbidity and mortality [1, 2]. DKD can be caused by high blood glucose, high blood pressure, or having diabetes for a long time. About 30–40% of diabetic patients will be complicated with DKD [3, 4], including glomerular hyperfiltration, progressive albuminuria, declining GFR, and ultimately leading to end-stage renal disease (ESRD) [5, 6]. Metabolic changes associated with diabetes lead to glomerular hypertrophy, glomerulosclerosis, and tubulointerstitial inflammation and fibrosis. Moreover, DKD patients often suffer from cardiovascular and neuropathy diseases, which sharply increases the risk of death [7, 8]. Although significant outcomes have been achieved in the pathogenesis and clinical therapy for DKD, there is a large residual risk of diabetic kidney disease onset and progression. Therefore, widespread innovation is urgently needed to improve health outcomes for patients with diabetic kidney disease. Achieving this goal requires further understanding molecular mechanism of DKD.

Recent attention has been focusing on the effect of senescence on lifespan and disease progression [9]. Senescence is a state of stable cell cycle arrest that occurs due to a variety of stimuli, such as DNA damage, oncogene activation, and telomere shortening [10]. Senescent cells are viable and metabolically active with other phenotypic alterations, playing roles in normal development, maintains tissue homeostasis, and limits tumor progression [11]. Senescent cells have several distinguishing characteristics, such as increased cell size and enzymatic activity of the lysosomal hydrolase senescence-associated β-galactosidase (SA-β-Gal), upregulation of prosurvival pathways to resist apoptosis, and the development of a “senescence-associated secretory phenotype (SASP)” [12]. The SASP is composed of a variety of soluble signaling factors, including pro-inflammatory cytokines, chemokines, and growth factors, insoluble extracellular matrix proteins, and non-protein components, which can be secreted into the surrounding microenvironment to affect neighboring cell biology and ultimately disrupting tissue structure and function [13]. Senescent cells are also characterized by an increase in the cyclin-dependent kinase inhibitor p16, which is one of the hallmarks of senescence [14]. It has been reported that purging p16-positive senescent cells in INK-ATTAC mice, a transgenic mouse model that allowed the removal of p16^Ink4a^-positive cells upon administration of a drug, AP20187, increased mean lifespan [9]. However, whether and how p16-positive senescent cells promote the progression of DKD remain unknown.

Recent studies support a key role of altered renal energy metabolism in the development of chronic kidney disease, including DKD [15, 16]. A range of metabolic abnormalities, including tricarboxylic acid circulation (TCA) disorders, altered fatty acid oxidation, and amino acid metabolic abnormalities, have been associated with kidney damage in DKD [17, 18]. However, the relationship between senescence and metabolic abnormalities in DKD and whether and how p16-positive senescent cells promote metabolic abnormalities and then DKD progression remains elusive. In this study, we address these questions by inducing diabetes in INK-ATTAC mice to determine the role of p16-positive senescent cells in kidney injury, glucolysis, and mitochondrial metabolic disorder in DKD.

## Results

### The expression of p16 is upregulated in the kidneys of DM mice and DKD patients

**Fig. 1:**
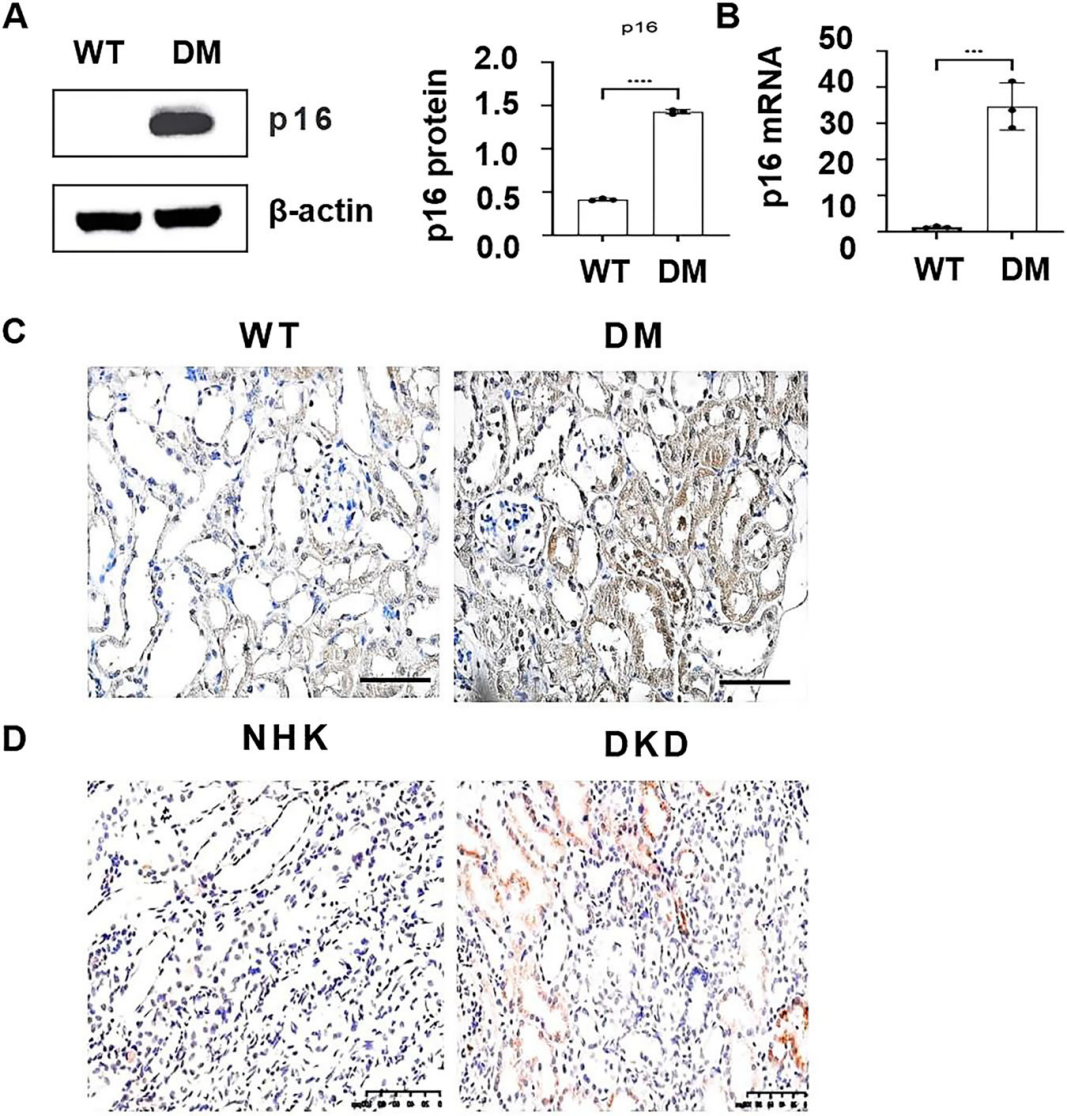
The expression of p16 is upregulated in DKD kidneys. **A** P16 protein and **B** mRNA levels in kidneys from diabetic (DM) mice and wild-type (WT) mice as analyzed through Western blot and qRT-PCR, respectively. ****p < 0.0001. *n* = 3. **C** Immunohistochemical staining of p16 in WT mice kidney tissues and DM mice kidney tissues. **D** Immunohistochemical staining of p16 in normal human kidney (NHK) tissues and kidneys from DKD patients. Scale bars: 100 μm.

To understand the role of p16 in DKD, first, we found that the expression of p16 was increased in kidneys from DM mice compared with age-matched wild-type mice, as analyzed with Western blot (Fig. 1A), quantitative reverse-transcription PCR (qRT-PCR) (Fig. 1B) and immunohistochemistry staining (Fig. 1C). The expression of p16 was also increased in kidneys from DKD patients compared with normal human kidneys as analyzed with immunohistochemistry staining (Fig. 1D), which was predominantly concentrated in renal tubular epithelial cells (TECs). This distribution of p16 in proximal tubules was further validated by its co-localization with lotus tetragonolobus lectin (LTL), a proximal tubular marker (Supplemental Fig. S1).

### Upregulation of p16 promotes senescence and increases the expression of the components of SASPs in the kidneys of DM mice and HK2 cells cultured in high glucose media

**Fig. 2:**
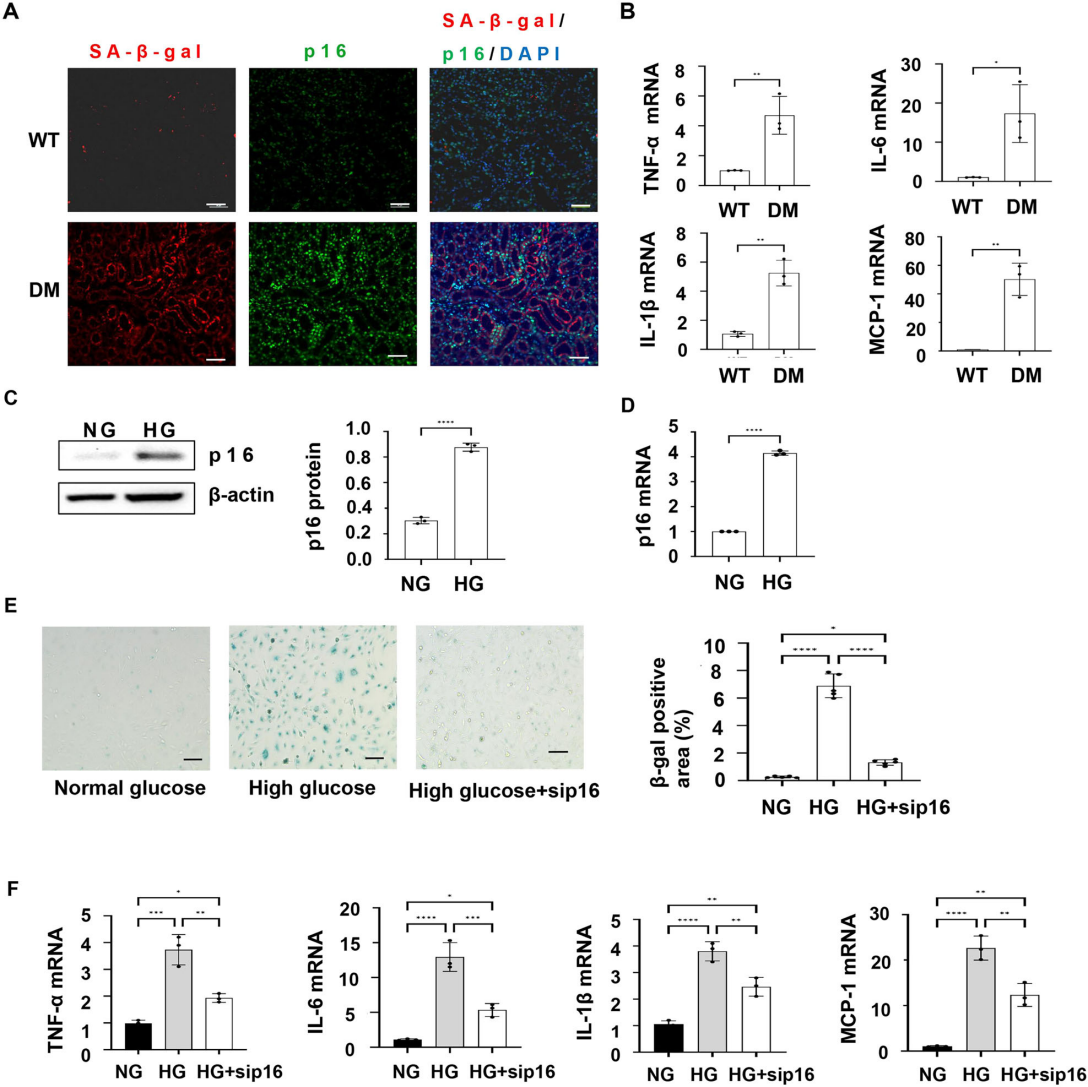
Upregulation of p16 promotes senescence and elevates the expression of the components of senescence-associated secretory phenotypes (SASPs). **A** Representative images of senescence-associated SA-β-gal (red) co-stained with p16 (green) in the kidneys of WT mice and DM mice. Scale bar: 50 μm. **B** qRT-PCR analysis of SASPs (TNF-α, IL-6, IL-1β, and MCP-1) mRNA in WT and DM mice kidneys. (*p < 0.05, **p < 0.01 as determined by one-way ANOVA). *n* = 3. **C** Western blot analysis of the protein levels of p16 in HK2 cells cultured in normal glucose medium (NG) and high glucose medium (HG). ****p < 0.0001. **D** qRT-PCR analysis of p16 mRNA level in HK2 cells cultured in normal glucose medium and high glucose medium. ****p < 0.0001. *n* = 3. **E** Senescence-associated SA-β-gal staining increased in HK2 cells cultured in normal glucose medium, high glucose medium, and high glucose medium with p16 knockdown. Scale bar: 50 μm. **F** qRT-PCR analysis of SASPs (TNF-α, IL-6, IL-1β, and MCP-1) mRNA in HK2 cells cultured in normal glucose medium, high glucose medium, and high glucose medium with p16 knockdown. *p < 0.05, **p < 0.01, ***p < 0.001, and ****p < 0.0001, as determined by one-way ANOVA. *n* = 3.

Upregulation of p16 has been used as one of the biomarkers of senescence [19]. In addition to an increase of p16-positive cells, we found that SA-β-gal positive cells was increased in the kidneys of DM mice as examined with the SA-β-gal staining (Fig. 2A). The acquisition of a senescence-associated secretory phenotype (SASP) is also a characteristic of senescent cells, and the secretion of SASPs can modify the cellular microenvironment and affect the function of neighboring cells [13]. We found that the expression of the most extensively characterized SASPs, such as TNF-α, IL-6, IL-1β, and MCP-1, was upregulated in kidneys from DM mice compared with age-matched wild-type control mice, as analyzed with qRT-PCR analysis (Fig. 2B).

To further understand the role of p16 in DKD, we cultured human kidney proximal tubular epithelial cells (HK2) with high glucose (25 mM) media to mimic a hyperglycemic (HG) condition in DKD. We found that treatment with high glucose (25 mM) increased the expression of p16 in HK2 cells compared to that in cells cultured in normal glucose (5 mM) media as analyzed with Western blot (Fig. 2C) and qRT-PCR (Fig. 2D). We also found that treatment with high glucose (25 mM) induced senescence of HK2 cells as seen by an increase of SA-β-gal staining (Fig. 2E) and the expression of SASPs, including TNF-α, IL-6, IL-1β, and MCP-1, compared to that in cells cultured in normal glucose media (Fig. 2F), whereas knockdown of p16 with siRNA decreased those effects in HK2 cells cultured in high glucose media. These results suggest that an upregulation of p16 may play a role in the progression of diabetic kidney disease through induction of senescence and the elevation of SASPs.

### Clearance of p16-positive senescent cells delays the progression of diabetic kidney disease

**Fig. 3:**
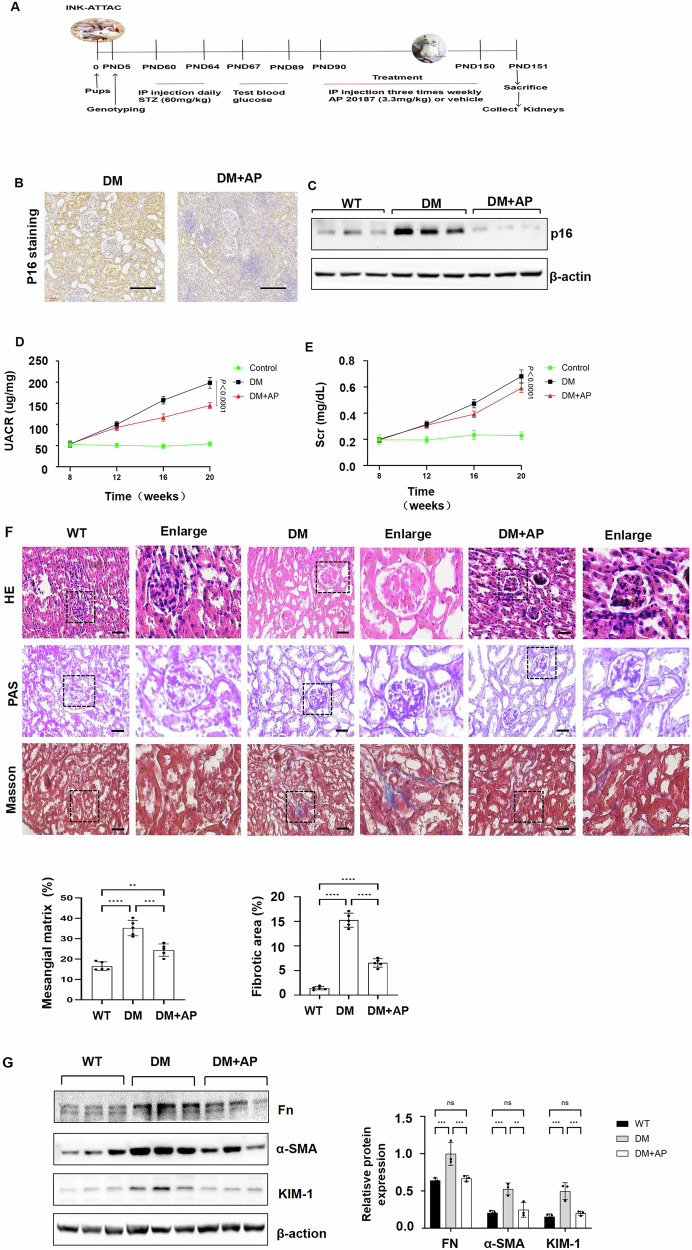
Clearance of p16-positive senescent cells delays the progression of DKD in mice. **A** Experimental design and timeline in INK-ATTAC transgenic mice were studied for 5 months. **B** Immunohistochemistry analysis indicated that p16 was decreased in AP20187-treated DM INK-ATTAC mice kidneys compared to vehicle-treated DM INK-ATTAC mice kidneys. Scale bar: 100 μm. **C** Western blot analysis of p16 expression in kidneys from WT, vehicle-treated DM INK-ATTAC, and AP20187-treated DM INK-ATTAC mice. **D**, **E** Urine microalbuminuria-to-creatinine ratio (UACR) and serum creatinine in WT, vehicle-treated DM INK-ATTAC, and AP20187-treated DM INK-ATTAC mice. Data represent the mean ± SEM for eight mice per group. **F** HE staining, PAS staining, and Masson trichrome staining in the kidneys from WT, vehicle-treated DM INK-ATTAC, and AP20187-treated DM INK-ATTAC mice. Scale bar: 100 μm. **G** Western blot analysis of the expression of fibronectin (Fn), a-SMA, and KIM-1 in WT, vehicle-treated DM INK-ATTAC, and AP20187-treated DM INK-ATTAC mice kidneys. *p < 0.05, **p < 0.01, and ***p < 0.001, as determined by one-way ANOVA.

Given that senescence and the expression of p16 were increased in kidneys of DM mice and DKD patients, it is reasonable to investigate whether clearance of p16-positive cells, most of them are senescent cells, decreases kidney injury and delays disease progression in DM mice. We induced type 1 diabetes in INK-ATTAC transgenic mice, a mouse model that allows the selective suicide gene-mediated ablation of highly p16-expressing cells upon administration of the drug AP20187 [9]. In brief, we treated 2-month-old INK-ATTAC male mice with low-dose streptozotocin (STZ) (60 mg/kg) and an equivalent volume of sodium citrate (vehicle control) by daily intraperitoneal injection for 5 days, which should induce persistent hyperglycemia in mice after 4 weeks, with the development of albuminuria. The DM INK-ATTAC transgenic mice were then treated intraperitoneally with AP20187 (3.3 mg/kg) and DMSO three times a week for 2 months, and kidneys were harvested one day after the last treatment with AP (Fig. 3A). Treatment with AP20187 cross-linked the ATTAC fusion protein, thereby activating its caspase-8 moiety to induce an apoptotic cell death, indicating as that the enhanced green fluorescence protein (EGFP) could not be detected after the induction with AP (Supplemental Fig. S2). We found that administration of AP20187 significantly decreased p16 level in the kidneys of DM INK-ATTAC mice compared to that in the kidneys from DMSO-injected age-matched DM INK-ATTAC mice, as analyzed with immunohistochemistry staining and Western blot analysis (Fig. 3B, C). Administration of AP20187 also decreased kidney weight/body weight ratios (KW/BW ratios), fasting blood glucose (Supplemental Fig. S3), urine microalbuminuria creatinine ratio (UACR), and serum creatinine (Fig. 3D, E) in DM INK-ATTAC mice compared to DMSO-treated DM INK-ATTAC mice. In addition, clearance of p16-positive cells with the treatment of AP20187 decreased glomerular matrix deposition, renal tubular atrophy and renal interstitial fibrosis in DM INK-ATTAC mice as examined by hematoxylin and eosin (H&E), periodic acid-Schiff (PAS) and Masson blue staining (Fig. 3F). The expression of kidney injury molecule-1 (KIM-1), a renal damage marker, and the expression of fibronectin and alpha-smooth muscle actin (α-SMA), two fibrotic markers, were also decreased in AP20187-treated DM INK-ATTAC kidneys compared to those in DMSO-treated DM INK-ATTAC kidneys (Fig. 3G). These results suggest that deletion of p16-positive senescent cells delays the progression of DKD.

### Clearance of p16-positive senescent cells releases cell arrest and alleviates kidney injury in diabetic kidneys through the Rb-CDK4 pathway and the decrease of SASPs

**Fig. 4:**
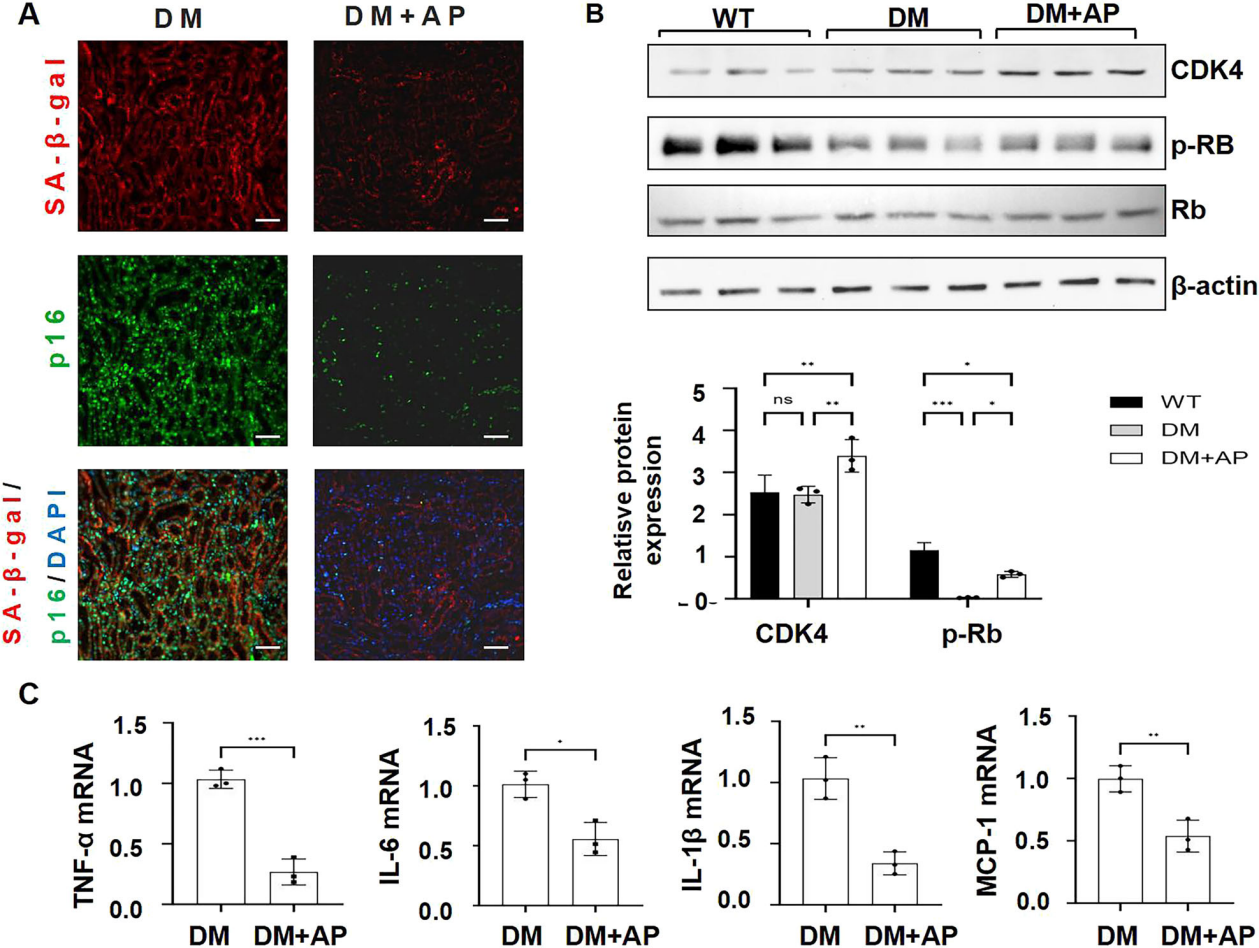
Clearance of p16-positive senescent cells alleviates kidney injury through the Rb-CDK4 pathway and the decrease of SASPs. **A** Representative images of senescence-associated SA-β-gal (red) co-stained with p16 (green) in kidneys from vehicle-treated DM INK-ATTAC mice and AP20187-treated DM INK-ATTAC mice. Scale bar: 50 μm. **B** Western blot analysis of the expression of CDK4, p-Rb, and Rb in WT, vehicle-treated DM INK-ATTAC, and AP20187-treated DM INK-ATTAC mice kidneys. The expression levels of CKD4 and p-Rb were decreased in kidneys from vehicle-treated DM INK-ATTAC mice compared to WT mice, and increased in AP20187-treated DM INK-ATTAC mice compared to vehicle-treated DM INK-ATTAC mice. **C** qRT-PCR analysis of SASPs (TNF-α, IL-6, IL-1β, and MCP-1) mRNA levels in kidneys from vehicle-treated DM INK-ATTAC mice and AP20187-treated DM INK-ATTAC mice. *p < 0.05, **p < 0.01, and ***p < 0.001, as determined by one-way ANOVA.

Senescent cells provoke permanent cell cycle arrest by CDK inhibitors [20], which can also mediate renal injury in DKD [21]. To confirm whether clearance of p16-positive cells alleviates senescence and to understand underling mechanism, first, we found that deletion of p16-positive cells decreased SA-β-gal staining in AP20187-treated DM INK-ATTAC kidneys compared to that in DMSO-treated DM INK-ATTAC kidneys (Fig. 4A). Second, we found that deletion of p16-positive cells increased the expression of CDK4 and the phosphorylation of Rb (Fig. 4B), which could inhibit E2F, a transcription factor, possibly resulting in a downregulation of the expression of genes in the regulation of cell proliferation [13]. Last, we found that deletion of p16-positive cells decreased the levels of SASPs, including IL-1β、IL-6、MCP-1, and TNF-α, in AP20187-treated DM INK-ATTAC kidneys as analyzed with qRT-PCR analysis (Fig. 4C), which might contribute to the downregulation of KIM-1 and other factors to decrease renal injury in those kidneys. These results suggest that p16-positive cells might block the cell cycle through the CDK4-pRb pathway in senescent cells and neighboring cells and promote renal injury via SASPs.

### Clearance of p16-positive cells normalizes adenosine triphosphate (ATP) contents, glycolysis, and impaired mitochondrial metabolism in AP20187-treated DM INK-ATTAC kidneys

**Fig. 5:**
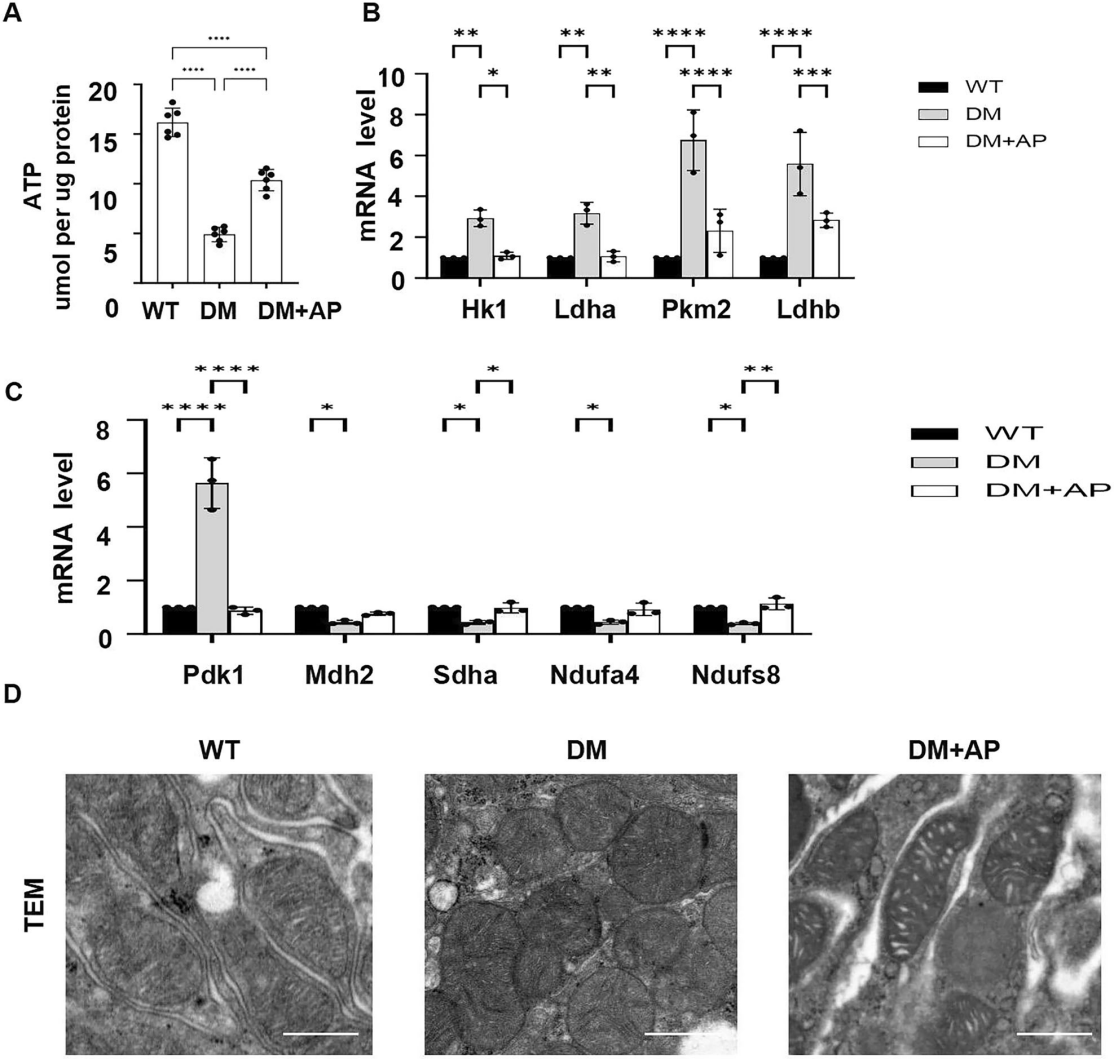
Clearance of p16-positive cells normalizes ATP contents, glycolysis, and impaired mitochondrial metabolism in DKD mouse kidneys. **A** ATP contents in kidneys from WT, vehicle-treated DM INK-ATTAC, and AP20187-treated DM INK-ATTAC mice. **B** qRT-PCR analysis of the mRNAs of the key glycolytic enzymes (*Hk1, Ldha, Pkm2, and Ldhb*) in kidneys of WT, vehicle-treated DM INK-ATTAC, and AP20187-treated DM INK-ATTAC mice. **C** qRT-PCR analysis of the expression of genes involved in mitochondrial metabolism, including *Pdk1, Mdh2, Sdha, Ndufa4,* and *Ndufs8*, in kidneys from WT, vehicle-treated DM INK-ATTAC, and AP20187-treated DM INK-ATTAC mice. **D** Representative transmission electron microscopy (TEM) images showing mitochondrial morphology in kidney sections from WT mice, vehicle-treated DM INK-ATTAC mice, and AP20187-treated DM INK-ATTAC mice. Scale bar: 1 mm. *p < 0.05, **p < 0.01, ***p < 0.001, and ****p < 0.0001 was determined by one-way ANOVA. ATP adenosine triphosphate.

Studies have suggested a functional link between altered renal energy metabolism and senescent phenotype in DKD [22]. In addition, glycolysis and mitochondrial metabolism play an important role in energy metabolism, and their dysregulation contributes to DKD progression [23]. We found that ATP contents were decreased in DM mouse kidneys compared to that in age-matched wild-type mouse kidneys, whereas deletion of p16-positive cells increased ATP contents in AP20187-treated DM INK-ATTAC kidneys (Fig. 5A). We further found that the expression of the key glycolytic enzymes, Hk1, Ldha, Pkm2, and Ldhb, was increased in DM mouse kidneys compared to that in age-matched wild-type mouse kidneys, whereas deletion of p16-positive senescent cells decreased the expression of these key glycolytic genes in AP20187-treated DM INK-ATTAC kidneys (Fig. 5B). In addition, the expression of the genes involved in mitochondrial metabolism, including Pdk1, Mdh2, Sdha, Ndufa4, and Ndufs8, was also altered in DM mouse kidneys compared to that in age-matched WT mouse kidneys, which could be normalized in AP20187-treated DM INK-ATTAC kidneys (Fig. 5C). We also observed mitochondrial swelling and altered cristae in kidneys of DM mice compared to those in kidneys of wild-type mice, whereas clearance of p16-positive senescent cells partial normalized mitochondrial morphology and cristae density in AP20187-treated DM INK-ATTAC lidneys as examined with transmission electron microscopy (TEM) (Fig. 5D). These results support that deletion of p16-positive cells can normalize energy metabolism and mitochondrial morphology in DKD kidneys.

### Knockdown of *p16* restored glycolysis and metabolism in HK2 cells

**Fig. 6:**
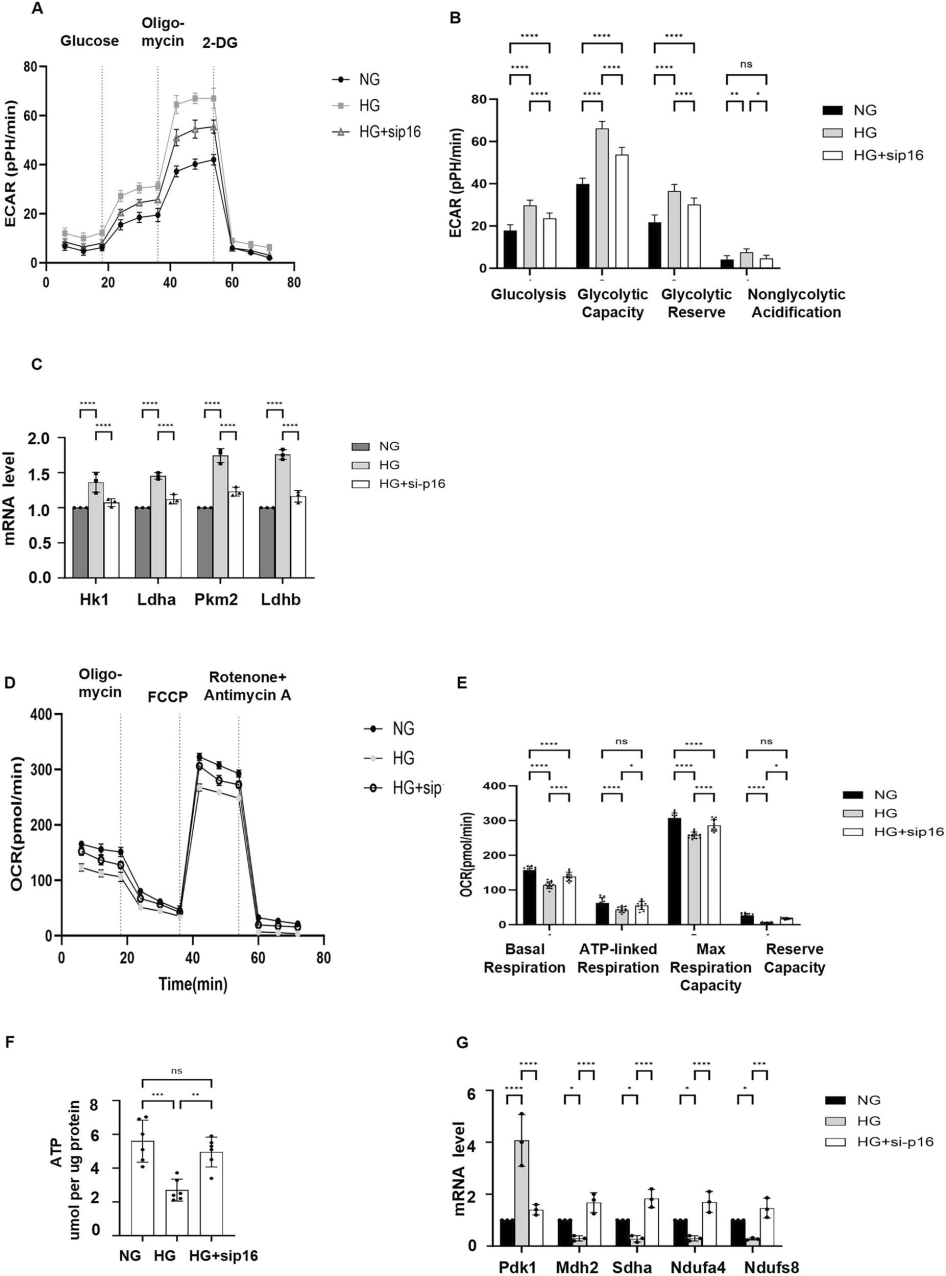
Knockdown of p16 normalizes ATP contents, glycolysis, and impaired mitochondrial metabolism in HK2 cells cultured in high glucose medium. **A** Glycolytic activity of HK2 cells cultured in normal glucose medium, high glucose medium, and high glucose medium with p16 knockdown was detected by extracellular acidification rates (ECARs). **B** Glycolysis, glycolytic capacity, glycolytic reserve, and nonglycolytic acidification were calculated based on the glycolysis stress tests. **C** qRT-PCR analysis of the mRNAs of the key glycolytic enzymes (*Hk1*, *Ldha*, *Pkm2*, and *Ldhb*) in HK2 cells cultured in normal glucose media, high glucose media, and high glucose medium with p16 knockdown. **D** Oxygen consumption rate (OCR) of HK2 cells cultured in normal glucose medium (NG), high glucose medium (HG), and high glucose medium with p16 knockdown (HG+sip16) were quantified. **E** The basal respiration, ATP-linked respiration, max respiration capacity, and reserve capacity were calculated based on the mitochondrial stress tests. **F** Intracellular ATP contents in HK2 cells cultured in normal glucose medium, high glucose medium, and high glucose medium with p16 knockdown were directly quantified by the ATP determination kit. **G** qRT-PCR analysis of the expression of genes involved in mitochondrial metabolism, including *Pdk1, Mdh2, Sdha, Ndufa4,* and *Ndufs8*, in HK2 cells cultured in normal glucose medium, high glucose medium, and high glucose medium with p16 knockdown. (“ns” implies no significant changes, *p < 0.05, **p < 0.01, ***p < 0.001, and ****p < 0.0001 was determined by one-way ANOVA. ATP adenosine triphosphate.

The in vivo analysis suggests that p16-positive cells plays an important role in the metabolic reprogramming in DKD. To further support this notion, we knocked down p16 with siRNA in HK2 cells cultured in high glucose (25 mM) media and then tested its effect on glycolysis and mitochondrial metabolism in those cells with Seahorse analysis. We found that knockdown of p16 increased glycolysis, maximal glycolytic capacity, and glycolytic reserve in HK2 cells cultured in high glucose media compared to HK2 cells cultured in normal glucose media (Fig. 6A, B). Consistently, knockdown of p16 decreased the expression of the key glycolysis genes, *Hk1, Ldha, Pkm2*, and *Ldh*, in HK2 cells cultured in high glucose media compared with HK2 cells cultured with normal glucose media (Fig. 6C). In addition, we found that the oxygen consumption rate (OCR) and ATP level were decreased in HK2 cells cultured in high glucose media compared to HK2 cells cultured in normal glucose media, indicating that overall mitochondrial respiration (basal respiration, ATP-linked respiration, maximal respiration and reserve capacity) was decreased in those cells cultured in high glucose media, whereas knockdown of p16 restored the oxygen consumption rate and ATP level in HK2 cells cultured in normal glucose media (Fig. 6D–F). Furthermore, we found that the expression of genes involved in mitochondrial metabolism was altered in HK2 cells cultured with high glucose, which could be normalized in p16 knockdown HK2 cells cultured with high glucose media (Fig. 6G). The results support a direct role of the upregulation of p16 in the regulation of glycolysis and energy metabolism in tubular epithelial cells in DKD kidneys.

### Knockdown of p16 decreases mitochondrial metabolic disorder through the AMPK and mTOR pathways

**Fig. 7:**
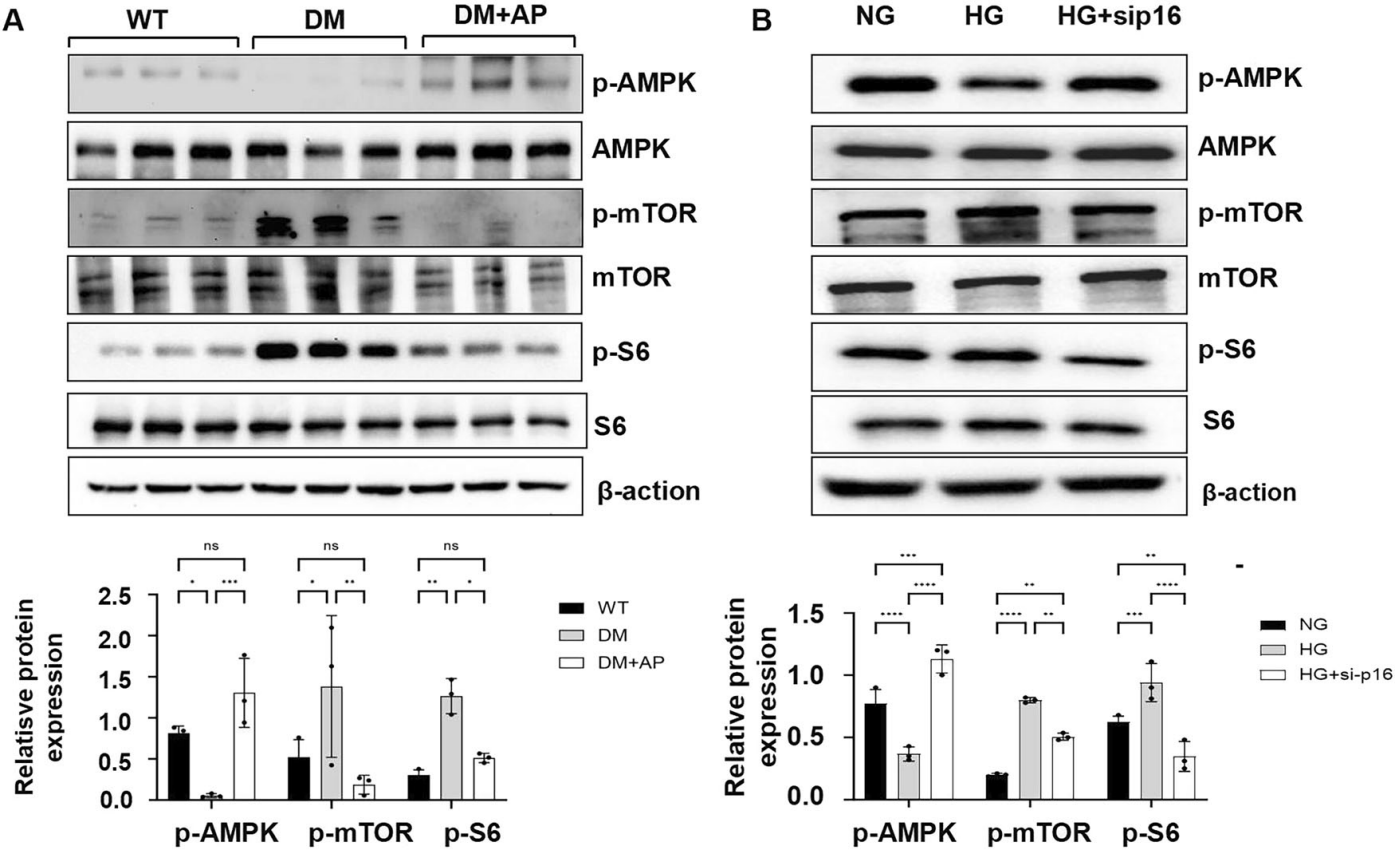
Knockdown of p16 decreases mitochondrial metabolic disorder through the AMPK and mTOR pathways. **A** Western blot analysis of the expression of p-AMPK, AMPK, p-mTOR, mTOR, p-S6, and S6 in WT, vehicle-treated DM INK-ATTAC, and AP20187-treated DM INK-ATTAC mice kidneys. The expression level of p-AMPK was decreased in kidneys from DM mice compared to WT mice, and increased in kidneys from DM with AP treatment mice compared to vehicle-treated DM mice. The expression levels of p-mTOR and p-S6 were increased in kidneys from DM mice compared to WT mice, and decreased in kidneys from DM with AP treatment mice compared to DM mice. **B** Western blot analysis of the expression of p-AMPK, AMPK, p-mTOR, mTOR, p-S6, and S6 in HK2 cells cultured in normal glucose medium (NG), high glucose medium (HG), and high glucose medium with p16 knockdown (HG + sip16). The expression level of p-AMPK was decreased in HK2 cells cultured in high glucose medium compared to normal glucose medium, and increased in HK2 cells from high glucose medium with p16 knockdown compared to high glucose medium. The expression levels of p-mTOR and p-S6 were increased in HK2 cells cultured in high glucose medium compared to normal glucose medium and decreased in HK2 cells from high glucose medium with p16 knockdown compared to high glucose medium. “ns” implies not significant, *p < 0.05, **p < 0.01, ***p < 0.001, and ****p < 0.0001 was determined by one-way ANOVA.

**Fig. 8:**
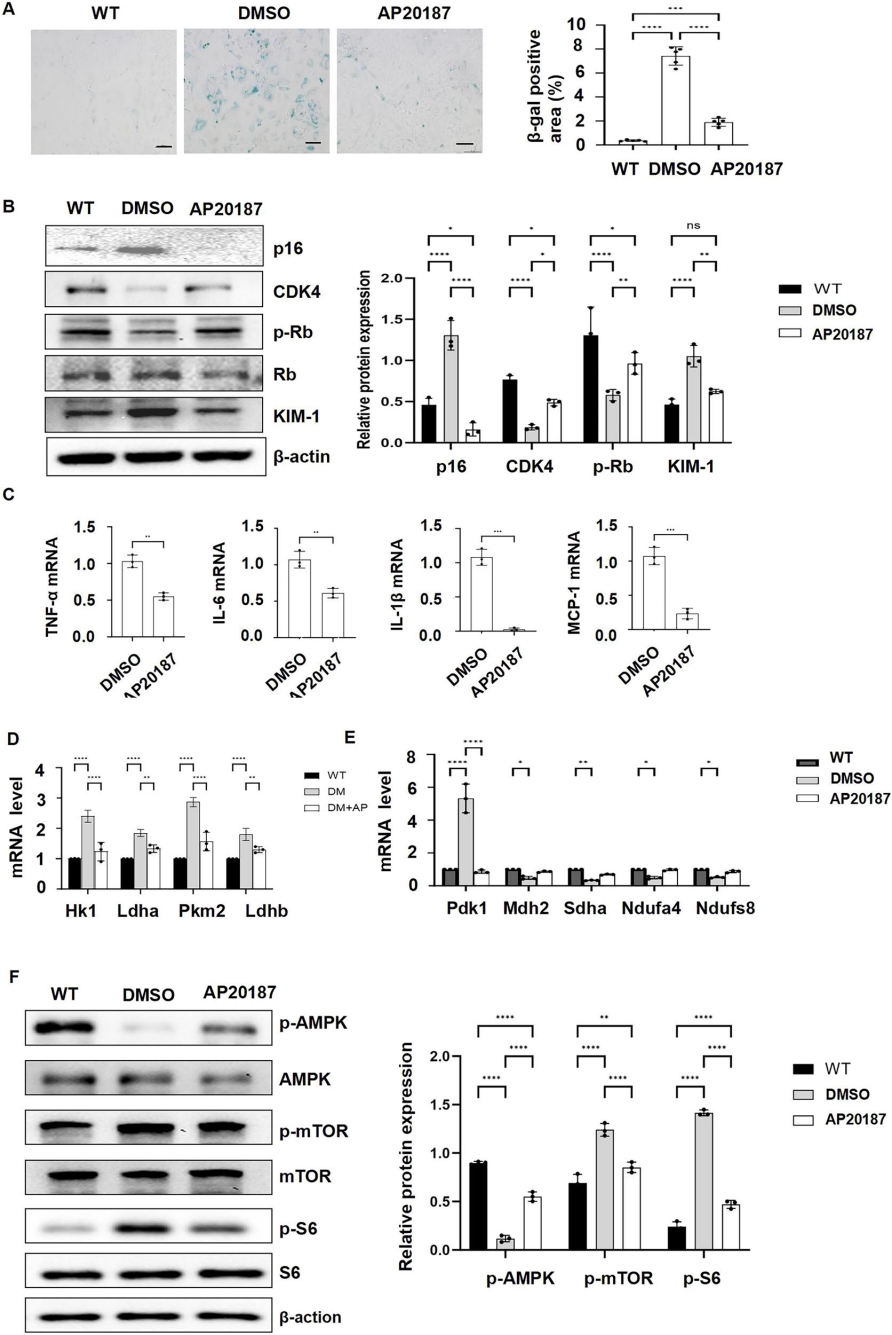
Deletion of p16-positive cells affects the neighboring cell biology and function in DKD. **A** Senescence-associated SA-β-gal staining in primary tubular epithelial cells (PTECs) from WT, vehicle-treated DM INK-ATTAC, and AP20187-treated DM INK-ATTAC mice. **B** Western blot analysis of the expression of p16, CDK4, p-Rb, Rb, and KIM-1 in PTECs from WT, vehicle-treated DM INK-ATTAC, and AP20187-treated DM INK-ATTAC mice. **C** qRT-PCR analysis of SASPs (TNF-α, IL-6, IL-1β, and MCP-1) mRNA levels in PTECs from vehicle-treated DM INK-ATTAC and AP20187-treated DM INK-ATTAC mice. **D** qRT-PCR analysis of the mRNAs of the key glycolytic enzymes (*Hk1, Ldha, Pkm2*, and *Ldhb*) in HK2 cells cultured in PTECs from WT, vehicle-treated DM INK-ATTAC, and AP20187-treated DM INK-ATTAC mice. **E** qRT-PCR analysis of the genes involved in mitochondrial metabolism, Pdk1, Mdh2, Sdha, Ndufa4, and Ndufs8, in PTECs from WT, vehicle-treated DM INK-ATTAC, and AP20187-treated DM INK-ATTAC mouse kidneys. **F** Western blot analysis of the expression of p-AMPK, AMPK, p-mTOR, mTOR, p-S6, and S6 in PTECs from WT, vehicle-treated DM INK-ATTAC, and AP20187-treated DM INK-ATTAC mice. “ns” implies not significant, *p < 0.05, **p < 0.01, ***p < 0.001, and ****p < 0.0001 was determined by one-way ANOVA.

Glycolytic metabolites have been reported to lead to mitochondrial metabolic disorder through AMPK and mTOR signaling [23]. We found that the phosphorylation of AMPK (active AMPK) was decreased in HK2 cells cultured with high glucose compared to that in HK2 cells cultured with normal glucose, whereas knockdown of p16 increased the phosphorylation of AMPK in HK2 cells cultured with high glucose (Fig. 7A). In addition, the phosphorylation of mTOR and its downstream targets, the ribosomal protein S6, were increased in HK2 cells cultured with high glucose compared with HK2 cells cultured with normal glucose, which could be decreased in p16 knockdown HK2 cells cultured with high glucose (Fig. 8A). We further found that the phosphorylation of AMPK was decreased and the phosphorylation of mTOR and S6 was increased in kidneys of DM mice compared with age-matched wild-type mice (Fig. 8B). Deletion of p16-positive senescent cells increased the phosphorylation of AMPK and decreased the phosphorylation of mTOR and S6 in AP20187-treated DM INK-ATTAC kidneys compared to those in vehicle-treated DM INK-ATTAC kidneys (Fig. 7B). These results suggest that p16-positive senescent cells may regulate mitochondrial metabolism disorder in DKD through AMPK and mTOR pathway.

### Deletion of p16-positive cells affects the neighboring cell biology and function in DKD

To further understand the effect of p16-positive cells on neighboring cell biology and function in DKD, we isolated the primary tubular epithelial cells (PTECs) from AP20187 and vehicle-treated DM INK-ATTAC kidneys. We found that (1) the percentage of SA-β-gal positive cells was higher and the levels of CDK4 and the phosphorylation of Rb were lower (Fig. 8A, B), (2) the expression of KIM-1 (Fig. 8B) and SASPs (Fig. 8C) was increased, (3) the expression of the key genes encoding glycolytic enzymes, *Hk1, Ldha, Pkm2*, and *Ldhb*, was increased (Fig. 8D), (4) the expression of genes involved in mitochondrial metabolism, including *Pdk1, Mdh2, Sdha, Ndufa4*, and *Ndufs8*, was altered (Fig. 8E), and (5) the phosphorylation of AMPK was decreased and the phosphorylation of mTOR and the phosphorylation of S6 was increased, in PTECs from DMSO-treated DM INK-ATTAC mouse kidneys compared to those in PTECs from AP20187-treated DM INK-ATTAC mouse kidneys (Fig. 8F). These results suggest that after the deletion of p16-positive cells from the diabetic mouse kidneys, the effect of p16-positive cells on PTECs was released.

## Discussion

**Fig. 9:**
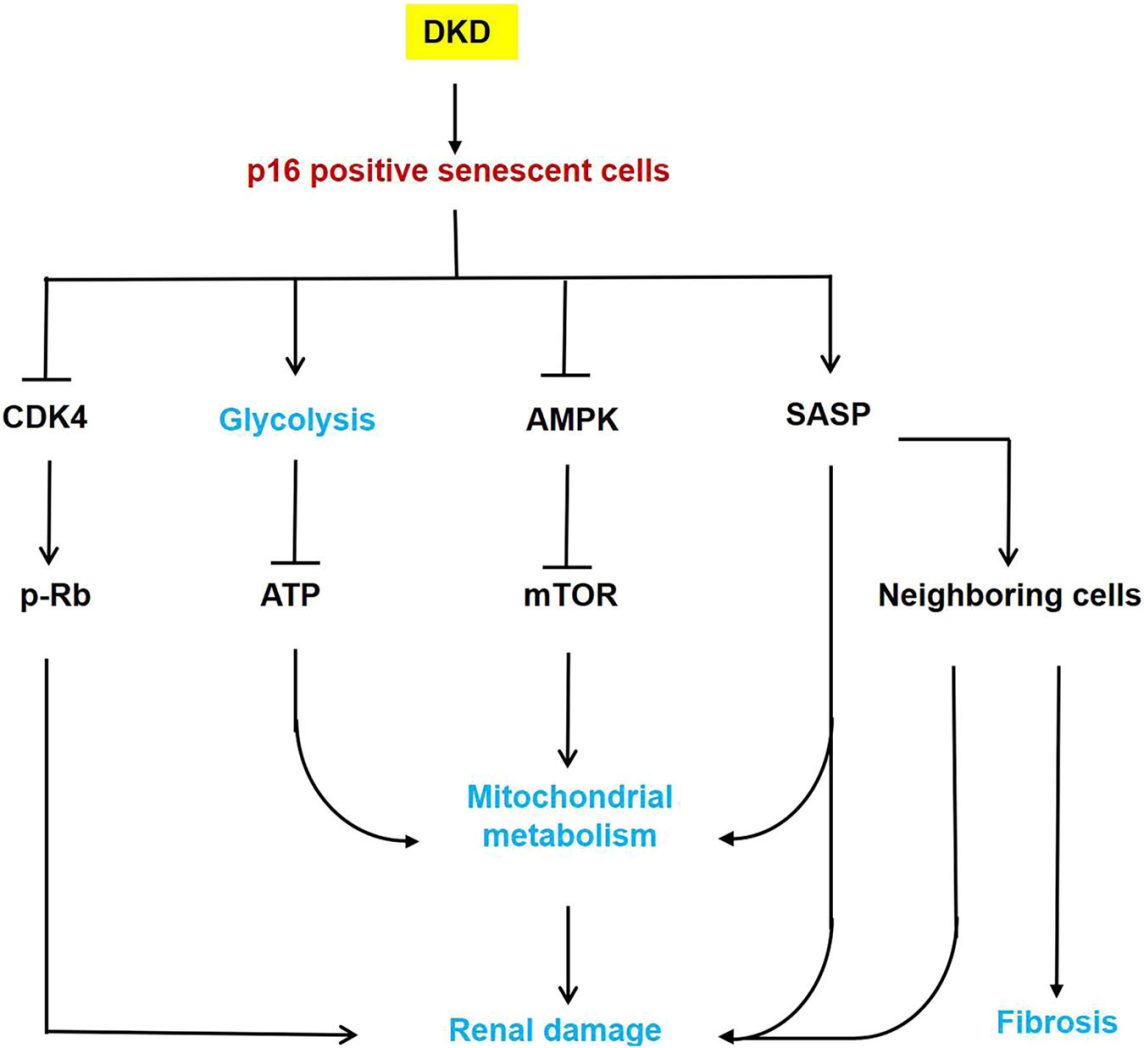
Working model of p16-positive senescent cells in DKD. A schematic representation of the roles and mechanisms of p16-positive senescent cells in the progression of DKD. Upregulated p16 in DKD kidneys, leading to an increase of senescence, resulted in (i) inhitbition of CDK4 and the phosphorylation of Rb, which could inhibit E2F, resulting in a downregulation of the expression of genes in the regulation of cell proliferation; (ii) increasing the genes of the key glycolytic enzymes and decreasing the production of ATP; (iii) abnormalizing the expression of the genes associated with mitochondrial metabolism, through AMPK and mTOR pathway; (iiii) increasing the expression of SASPs. They all promote the progression of diabetic kidney disease.

DKD is a leading cause of kidney failure in the world, representing ~44% of all cases of kidney failure in the United States [24, 25], and new interventions are needed to reduce the health burden of DKD [26]. In this study, we show that p16-positive senescent cells are a key regulator of DKD. The expression of p16 was upregulated in DKD kidneys and HK2 cells cultured in high glucose media, resulting in an increase of senescence and the expression of SASPs, including TNF-α, IL-6, IL-1β, and MCP-1, to decrease the production of ATP and promote glycolysis and mitochondrial metabolic disorder. Clearance of p16-positive cells delayed the progression of diabetic kidney disease in STZ-induced INK-ATTAC transgenic mice, restored the levels of ATP, decreased the expression of the common SASPs, decreased glycolysis, and improved metabolic reprogramming of mitochondria, possibly through the AMPK and mTOR pathways (Fig. 9). This study suggests that pharmacological deletion of p16-positive senescent cells may be a novel therapeutic strategy for DKD treatment.

Growing evidence indicates that an accumulation of senescent cells is associated with the loss of kidney function in DKD [27]. In diabetic kidneys, senescence is induced by multiple stressors, including high blood glucose, accumulation of advanced glycation end products (AGE), hypertension, oxidative stress, and inflammation, resulting in a decline of renal function and changes in renal structures [28]. One of the key features of this study is to determine the direct role of p16-positive senescent cells in DKD by using the INK-ATTAC transgenic mouse model. The INK-ATTAC mouse has been generated by researchers at the Mayo Clinic [9], which includes a 2617 bp fragment of the promoter of the p16 INK4A gene and an internal ribosome entry site (IRES), followed by an open reading frame (ORF) encoding enhanced green fluorescent protein (EGFP) to allow detection of p16 INK4A-positive senescent cells with the treatment of drug AP20187 [9]. AP20187 is a synthetic drug that induces dimerization of the membrane-bound myristylated FK506-binding-protein-caspase-8 (FKBP-Casp8) fusion protein via the p16 promoter to selectively induce p16-positive senescent cell death in INK-ATTAC mice [9]. We induced type 1 diabetes in the INK-ATTAC transgenic mice and found that clearance of p16-positive senescent cells with the treatment of AP20187 alleviated kidney injury and the progression of DKD, which supports a direct role of p16-positive senescent cells in DKD.

The second key feature of this study is to determine how p16-positive senescent cells promote the progression of DKD. Our results suggest two potential mechanisms in this process. First, the increase of p16-positive senescent cells alters the expression of key enzymes involved in glycolysis and mitochondrial metabolism. Second, p16-positive senescent cells may affect the biology and function of neighboring cells through the secretion of SASPs, such as TNF-α, IL-6, IL-1β, and MCP-1, in a paracrine manner [28]. This may explain how a relatively small number of p16-positive senescent cells promotes DKD progression. To support the hypothesis that SASPs have potent local and systemic effects on neighboring normal cells, we isolated PTECs from vehicle and AP20187-treated DM INK-ATTAC kidneys and found that after the deletion of senescent cells from the diabetic mouse kidneys, the biology and function of the neighboring PTECs were normalized in DM INK-ATTAC kidneys compared to vehicle-treated controls.

Senescent cells secreted SASPs contain complement proteins, pro-inflammatory cytokines, and pro-fibrotic or pro-clotting factors [29]. These factors act either alone or together to affect renal cell biology and function, leading to pathological changes in different types of renal cells to promote renal tubulointerstitial fibrosis, podocyte hypertrophy and loss of foot process, mesangial stroma dilation, glomerular sclerosis, and glomerular hypertension [30]. Clearance of p16-positive senescent cells not only prevented cell cycle arrest but should also reduce SASP secretion via the decrease of their expression in diabetic mice, resulting in systemic benefits, including lower blood glucose. The beneficial effect of AP20187 treatment on alleviating DKD injury may also be attributed to the reduction of blood glucose mediated by SASPs. These effects may also be related to the elimination of senescent cells in adipose tissues, thereby improving glucose-induced insulin secretion and glucose tolerance in DKD kidneys [29, 31].

The third key feature of this study is that p16-positive senescent cells can affect ATP production and energy metabolism in DKD kidneys. DKD is characterized by abnormal kidney energy metabolism, but the exact mechanism is unclear. Recent studies have demonstrated that abnormal glucose metabolism in DKD is mainly manifested by increased glycolysis and elevated lactate level, which are associated with albuminuria and kidney injury [32]. In tumor cells, even when oxygen levels are normal, glycolysis is prioritized to produce energy, known as the Warburg effect [33]. However, in DKD, the increase of renal glycolysis is mainly attributed to the obstruction of tricarboxylic acid circulation, resulting in impaired oxidative phosphorylation and ATP production [34, 35]. The enhanced glycolytic activity reveals elevated glycolysis in HK2 cells cultured in high glucose medium. Glycolysis increases the production of lactic acid by producing pyruvate, which is associated with the upregulation of lactate dehydrogenase (LDH), pyruvate kinase (PKM), and hexokinase (Hk1) [36]. PKM is responsible for the last conversion step of glycolysis to produce pyruvate [37]. PKM is also thought to indirectly increase lactic acid production [38]. Meanwhile, the upregulation of LDH results in an increase of lactate levels in senescent cells [39], leading to multiple senescence-associated events, such as tumorigenesis, wound healing, and evasion of immune responses [40]. Clearance of p16-positive senescent cells not only increases ATP levels but also normalizes the expression of key enzymes in glycolysis, including hexokinase, lactate dehydrogenase, and pyruvate kinase in DKD kidneys, suggesting that an improvement of energy and glucose metabolism is a critical benefit of clearing p16-positive senescent cell therapy in DKD.

The fourth key feature of this study is that p16-positive senescent cells can affect mitochondrial function in DKD kidneys. Increasing studies revealed the role of mitochondrial dysfunction in the pathogenesis of DKD [41]. Insulin resistance and other related metabolic disorders in DKD may damage mitochondria and limit their ability to select glucose as the preferred substrate for oxidation, known as metabolic inflexibility [18, 41]. The primary biological function of mitochondria is responsible for generating a substantial amount of ATP, thereby supplying the energy source for basal cell functions in the kidneys [39]. ATP production in mitochondria is accomplished by a continuous reaction called oxidative phosphorylation (OXPHOS), and changes in OXPHOS function have been observed in various senescent models [39, 42, 43]. During senescence, dysfunctional mitochondria produce excess oxidative stress (ROS), leading to unnecessary oxidation of the proteins involved in OXPHOS and impacting their function [44]. Changes in mitochondrial metabolism, with reduced dependence on OXPHOS but increased dependence on glycolysis, are one of the characteristic changes observed during senescence [45].

Mitochondrial glucose metabolism is regulated by the mTOR pathway and its upstream regulator, AMPK [46]. It has been reported that chronic hyperglycemia can lead to an inhibition of pyruvate dehydrogenase (PDH) through mTOR-S6 signaling, which is triggered by glycolytic metabolites upstream of glyceraldehyde-3-phosphate dehydrogenase (GAPDH) [23]. In DKD, the activity of AMPK is decreased and the activity of mTOR is increased, resulting in altered expression of genes involved in mitochondrial metabolism [47]. Clearance of p16-positive cells increases the activity of AMPK and decreases the activity of mTOR, leading to a normalization of the expression of mitochondrial metabolic genes, including the downregulation of Pdk1 (encoding pyruvate dehydrogenase kinases 1) in DKD kidneys. Pdk1 can phosphorylate PDH to inhibit its activity, in which the PDH complex is able to convert pyruvate to acetyl-CoA bin the mitochondria [48]. Thus, downregulation of Pdk1 mediated by the deletion of p16-positive senescent cells should restore pyruvate dehydrogenase activity and increase pyruvate entry into the tricarboxylic acid cycle in DKD kidney, which should be one of the mechanisms to decrease DKD progression.

Last, during normal conditions, the complex of CDK4 and CDK6, as well as cyclin D, phosphorylates Rb to promote the transition of cells from the G1 phase to S phase [49]. When p16 is elevated, it competes with cyclin D for the binding of CDK4, thereby decreasing the phosphorylation of Rb and S-phase entry [50]. We found that the expression of p16 was mainly increased in renal tubules but not in glomeruli in DKD kidneys, which should promote senescence of p16-positive tubular epithelial cells. Tubular and interstitium accounts for more than 90% of renal parenchyma with important functions. It has been reported that tubular structural abnormalities predate glomerular structural changes in DKD kidneys and play an important role in the progression of DKD [51]. However, the underlying mechanisms need to be further investigated. In addition, although treatment with high glucose could increase the expression of p16 and senescence in HK2 cells, it remains to be determined how high glucose regulates the expression of p16 in DKD kidneys.

## Methods

### Cell lines and primary cells

Human kidney proximal tubular epithelial cells (HK2, ATCC, Cat# CRL-2190) were cultured in DMEM/F12 (Hyclone, Logan, UT, USA) supplemented with 10% FBS (Sigma, St. Louis, MO, USA) and 1% penicillin-streptomycin (Gibco, Grand Island, NY, USA) at 37 °C in 5% CO_2_ incubator. The cells cultured in 5 mM glucose were named the NG group, and those cultured in 25 mM glucose were named the HG group. Primary tubular epithelial cells (PTECs) were isolated from AP20187 and vehicle-treated DM INK-ATTAC mice. The renal cortices were chopped and digested with 1 mg/ml collagenase II in PBS at 37 °C for 10 min. DMEM-F12 supplemented with 10% FBS were used to stop digestion. The mixture was passed orderly through a 100-μm and 70-μm filter (BD Falcon). The filtrate was collected at the bottom and centrifuged. The cells were seeded on a culture dish in DMEM-F12 supplemented with 10% FBS and 1% (v/v) penicillin-streptomycin.

### Western blot analysis

Cells were homogenized in lysis buffer (20 mM Tris-HCl, pH 7.4, 150 mM NaCl, 10% glycerol, 1% Triton X-100, 1 mM Na_3_VO_4_, 25 mM β-glycerol-phosphate, 0.1 mM PMSF, Roche complete protease inhibitor set, and Sigma-Aldrich phosphatase inhibitor set, St. Louis, MO, USA), and centrifuged at 20,000×*g* for 20 min. Protein concentration was measured using the BCA Pierce Protein assay kit (Thermo Fisher, Waltham, MA, USA) and normalized to the lowest concentration. Protein samples were subjected to standard SDS-PAGE gels, transferred to immuno-blot PVDF membranes (Millipore, Burlington, MA, USA), blocked with 10% nonfat dry milk, and then incubated overnight at 4 °C with primary antibodies. Primary antibodies used in this study are listed as follows: mouse monoclonal antibodies against CDKN2A/p16 (F12, Santa Cruz, Dallas, TX, USA, sc-1661, 1:500 dilution), α-SMA (Cell Signaling Technology, Boston, MA, #69313, 1:1000 dilution), Rb (CST, #9309, 1:1000 dilution), CDK4 (3F121, sc-70831, 1:1000 dilution), β-actin (AC-15, Sigma, St. Louis, MO, USA, A1978, 1:3000 dilution); rabbit polyclonal antibodies against beta Galactosidase (Invitrogen, A-11132, 1:300 dilution), Fibronectin/FN1 (CST, #36779, 1:1000 dilution), Phospho-Rb (CST, #8180, 1:1000 dilution), Phospho-AMPKα (Thr172) (CST, #2531, 1:1000 dilution), AMPKα (D5A2, CST, #5831, 1:1000 dilution), Phospho-mTOR (Ser2448) (D9C2, CST, #5536, 1:1000 dilution), mTOR (7C10, CST, #2983, 1:1000 dilution), Phospho-S6 ribosomal protein (Ser240/244) (D68F8, CST, #5364, 1:1000 dilution), S6 ribosomal protein (5G10, CST, #2217, 1:1000 dilution). rabbit monoclonal antibodies anti-KIM-1 (9E1, Abcam, Waltham, MA, USA, ab302932, 1:1000 dilution). Secondary antibodies used for Western blot include donkey anti-rabbit IgG–horseradish peroxidase (sc-2313) and goat anti-mouse IgG–horseradish peroxidase (sc-2005), purchased from Santa Cruz Biotechnology Inc. (Dallas, TX, USA).

### Histology and immunohistochemistry

Kidney sections were fixed in 4% paraformaldehyde (pH 7.4), and stained with hematoxylin and eosin (H&E), periodic acid-Schiff (PAS), and Masson trichrome staining methods. Immunohistochemistry was performed in 4-mm paraffin sections. For p16 staining, a monoclonal mouse anti-CDKN2A/p16 antibody (F12, Santa Cruz, Dallas, TX, USA, sc-1661, 1:100 used for immunohistochemistry [IHC]), biotinylated secondary antibody (Santa Cruz Biotechnology Inc., 1:500), and DAB substrate system were used. Kidney sections were counterstained by haematoxylin. The exposure time of the slides to the reagents was the same for the samples of all groups in each independent experiment. Images were analyzed with a NIKON ECLIPSE 80i microscope. The fibrotic area was quantified using Image J software (https://imagej.net/ij/, accessed on 5 June 2024) by Masson trichrome staining in ten random fields (original magnification ×200) as previously described [52].

### Immunofluorescence staining

After antigen retrieval, tissue sections were incubated with a mouse monoclonal antibody CDKN2A/p16 (F12, Santa Cruz, Dallas, TX, USA, sc-1661, 1:100 dilution) overnight, and then were incubated with Fluro-555 anti-mouse IgG secondary antibody or Fluro-488 anti-mouse IgG secondary antibody and mounted in Prolong Gold Antifade reagent with DAPI (Invitrogen). For beta galactosidase staining, a rabbit against beta galactosidase antibody (SA-β-gal Invitrogen, A-11132, 1:300 dilution) and Fluro-555 anti-rabbit IgG secondary antibody were used. Images were analyzed using a NIKON ECLIPSE 80i microscope.

### Quantitative reverse-transcription PCR (qRT-PCR)

Total RNA was extracted using the RNeasy Plus Mini Kit (QIAGEN, Germantown, MD, USA). Total RNA (1 μg) was reverse transcribed using an iScript cDNA Synthesis Kit (Bio-Rad, Hercules, CA, USA) and amplified in triplicate using iTaq SYBR Green Supermix with ROX (Bio-Rad) with a real-time PCR machine (Bio-Rad), according to the manufacturer’s instructions. Genes were amplified using the following primers: Hk1-F, 5′-TCACATTGTCTCCTGCATCTC-3′; Hk1-R, 5′-CTTTGAATCCCTTTGTCCACG-3′; Ldha-F, 5′-GCTCCCCAGAACAAGATTACAG-3′; Ldha-R, 5′-TCGCCCTTGAGTTTGTCTTC-3′; Pkm2-F, 5′-CCATTCTCTACCGTCCTGTTG-3′;

Pkm2-R, 5′-TCCATGTAAGCGTTGTCCAG-3′; Ldhb-F, 5′-TACGTCACCTGGAAACTGAG-3′; Ldhb- R, 5′-CACCATCTTATGCACCTCCT-3′

Pdk1-F, 5′-TCCCCCGATTCAGGTTCAC-3′; Pdk1-R, 5′-CCCGGTCACTCATCTTCACA-3′

Sdha-F, 5′-GAGATACGCACCTGTTGCCAAG-3′; Sdha-R, 5′-GGTAGACGTGATCTTTCTCAGGG-3′

Mdh2-F, 5′-GCAACCCCTTTCACTCCTG-3′; Mdh2-R, 5′-TCTGGTCTCAATGTGACTCAGAT-3′

NDUFA4-F, 5′-TCCCAGCTTGATTCCTCTCTT-3′; NDUFA4-R, 5′-GGGTTGTTCTTTCTGTCCCAG-3′; Ndufs8-F, 5′-TTGCCTGCAAACTCTGTGAG-3′; Ndufs8-R, 5′-CTCCACAATGGCATCAACAG-3′ ; β-actin-F, 5′-AAGAGCTATGAGCTGCCTGA-3′; β-actin-R, 5′-TACGGATGTCAACGTCACAC-3′. The complete reactions were subjected to the following program of thermal cycling: 40 cycles of 10 s at 95 °C and 20 s at 61 °C. A melting curve was run after the PCR cycles, followed by a cooling step. Each sample was run in triplicate in each experiment, and each experiment was repeated three times. Expression levels of target genes were normalized to the expression level of β-actin.

### Senescence-associated beta-galactosidase (SA-β-gal) staining

Renal cell senescence was evaluated using the Senescence Cells β-Galactosidase Staining Kit (#9860-Cell Signaling Technology) according to the manufacturer’s protocol. Early passage HK2 cells or primary tubular epithelial cells were washed in 1x PBS and fixed for 15 min (1x fixative solution) and then rinsed in 1x PBS two times. Add 1 ml of theβ-Galactosidase staining solution (1 ml containing 50 ul of 20 mg/ml X-gal stock solution, 930 µl of 1x Staining solution, 10 ul of 100x solution A, 10 ul of 100x solution B). Incubate the plate at 37 °C at least overnight in a dry incubator (no CO_2_). The development of blue dots/areas was counted as positive staining.

### Renal function measurement

Mice were placed in metabolic cages for the collection of 24-h urinary samples every 4 weeks from the age of 8 weeks. Urinary microalbumin was measured by competitive ELISA according to the manufacturer’s instructions (Exocell, Philadelphia, PA). Urinary and serum creatinine was measured by an enzymatic kit (Stanbio Laboratories, Boerne, TX). Urinary albumin excretion was expressed as total urinary albumin–to–creatinine ratio (mg/mg), as previously reported [53].

### Fasting blood glucose

Blood glucose levels were measured every 4 weeks by an AccuChek glucose meter (Roche Diagnostics, Basel, Switzerland) after the mice were fasted for 6 h, as recommended by the Animal Models of Diabetic Complications Consortium.

### RNA interference

The RNA oligonucleotides that specifically targeted human p16 (Cat# 4392420) were purchased from Thermo Fisher. The RNA oligonucleotides were transfected with DharmaFECT siRNA transfection reagent (GE Healthcare). Twenty-four hours after transfection, cells were harvested and analyzed by Western blotting and quantitative RT-PCR (qRT-PCR).

### ATP assay

The cells were transfected with p16 siRNA for 48 h and lysed with RIPA buffer. The intracellular ATP levels of cell lysates were assessed using the ATP Determination Kit (Invitrogen), according to the manufacturer’s protocol. The intracellular ATP levels were shown by normalizing to the protein amount.

### Mouse strain and treatment

All animal procedures were conducted under IACUC protocol A00003756-R24 (26 July 2024), approved by the Mayo Clinic IACUC, and in compliance with National Institutes of Health, United States Department of Agriculture, and Association for Assessment and Accreditation of Laboratory Animal Care guidelines. INK-ATTAC transgenic mice, which were used for studying renal senescence in vivo, were provided by J. van Deursen at the Mayo Clinic. These INK-ATTAC mice were bred onto a C57BL/6 genetic background as heterozygotes and genotyped. All mice were randomized to each group. Unless otherwise specified, the number of mice in each group is 8. Hyperglycemia was induced in 2-month-old INK-ATTAC male mice using low-dose streptozotocin (STZ) injection following established protocols (intraperitoneally, 60 mg/kg body weight, freshly dissolved in 0.05 mol/l sterile sodium citrate, pH 4.5 for 5 consecutive days) [54, 55]. One month later, mice which were induced to have persistent hyperglycemia were randomized to the treatment of AP (eight mice) or vehicle (eight mice). Age-matched control male mice received sodium citrate. AP (B/B Homodimerizer; Clontech, Mountain View, CA) leads to dimerization and then activates FKBP-fused caspase-8 components, leading to apoptosis of cells highly expressing p16Ink4a. AP only affects cells with the ATTAC fusion protein. AP (3.3 mg/kg) was delivered by intraperitoneal injections, three times a week, for 8 weeks (total 80 mg/kg, 24 treatments).

If an improper intraperitoneal injection operation causes the death of the mouse, exclude and supplement the same amount.

### Metabolic assays

The Agilent Seahorse XF mito stress test and XF glycolysis stress test were performed according to the manufacturer’s instructions. Cells were counted, and the same number of cells were plated per well in the Seahorse experiments. In addition, cells were examined microscopically for adherence to the culture plates immediately before Seahorse quantifications. Under basal conditions and following the addition of 10 mM glucose, 1 μM oligomycin, and 50 mM 2-DG, the extracellular acidification rates (ECAR) levels were measured. Oxygen consumption rate (OCR) were measured at the basal level and the addition of 1.5 μM oligomycin, 1 μM FCCP, and 0.5 μM Rot + AA.

### Statistical analysis

All of the data were expressed as mean ± SEM. Statistical analyses were performed with one-way ANOVA, followed by Newman–Keuls multiple comparison from GraphPad Prism 10.0 (GraphPad Software, La Jolla, CA). In addition, a repeated-analysis ANOVA was used for albumin excretion, body weight, and fasting blood glucose analysis.

## Supplementary information

Supplemental figure

Full and uncropped western blots

## Data Availability

The data used and analyzed in this study are available from the corresponding authors upon reasonable request.
