# Supplementary material for: P16-positive senescent cells promote DKD by the dysregulation of glycolysis and mitochondrial metabolism
Source: Cell Death Discov. 2025 Jul 30;11:355. doi: 10.1038/s41420-025-02650-2 (PMC12311014; doi:10.1038/s41420-025-02650-2)

### **Supplemental figure legends**

**Supplemental Figure S1. The distribution of p16 in kidneys of WT and DM mice.** Colocalization of p16 (red) with LTL (green), a marker of proximal tubular epithelial cells, in the kidney tissues. Scale bar, 50 µm.

**Supplemental Figure S2.** (A) The INK-ATTAC mouse has the enhanced green fluorescence protein (GFP) in kidneys. (B) Treatment with AP20187 induce an apoptotic cell death in kidneys. Scale bar, 50 µm.

**Supplemental Figure S3.** Administration of the durg AP20187 significantly decreased the (A) kidney weight/body weight ratios (KW/BW ratios) and (B) fasting blood glucose in DM INK-ATTAC mice compared to that in vehicle treated DM INK-ATTAC mice.


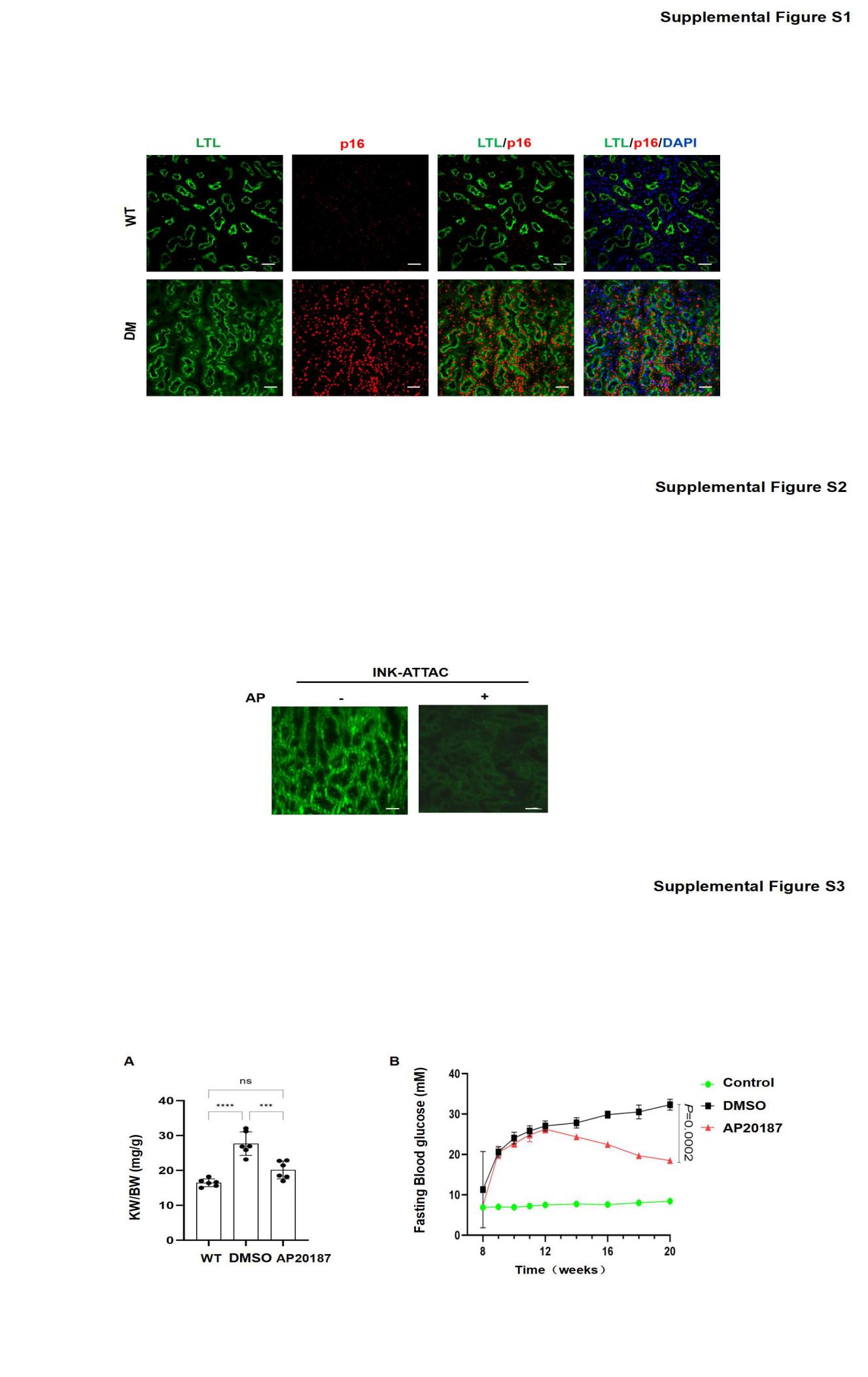

Supplement: Supplementary file 1 — Supplemental figure [file 41420_2025_2650_MOESM1_ESM.docx]
