# Supplementary material for: P16-positive senescent cells promote DKD by the dysregulation of glycolysis and mitochondrial metabolism
Source: Cell Death Discov. 2025 Jul 30;11:355. doi: 10.1038/s41420-025-02650-2 (PMC12311014; doi:10.1038/s41420-025-02650-2)
Supplement: Supplementary file 2 — Full and uncropped western blots [file 41420_2025_2650_MOESM2_ESM.pdf]

Figure 1A

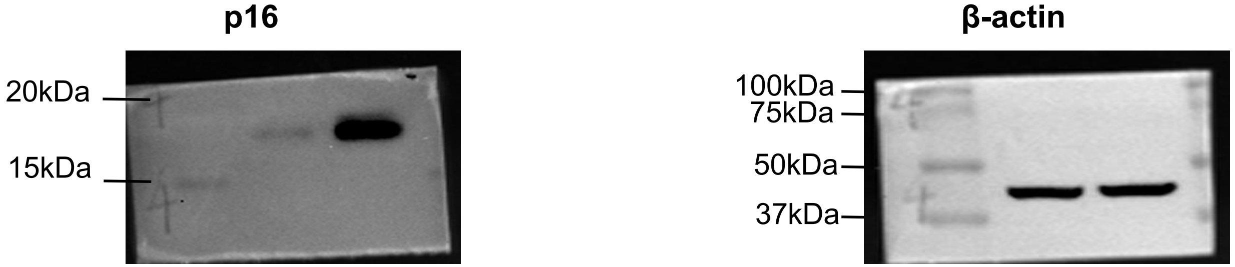

Figure 2C

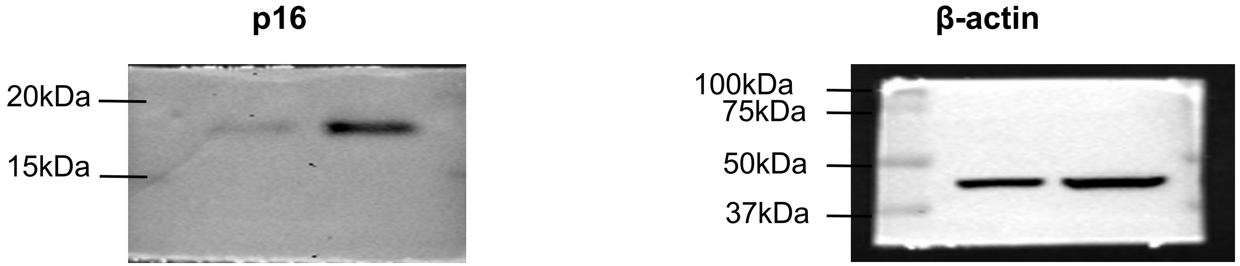

Figure 3C

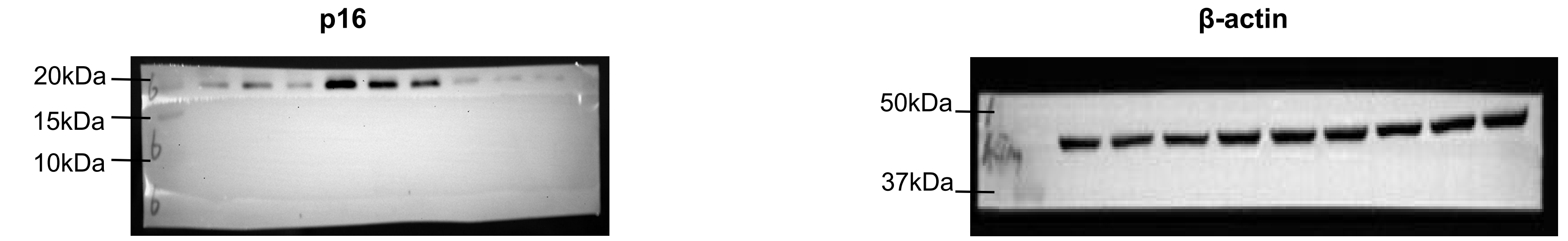

Figure 3G

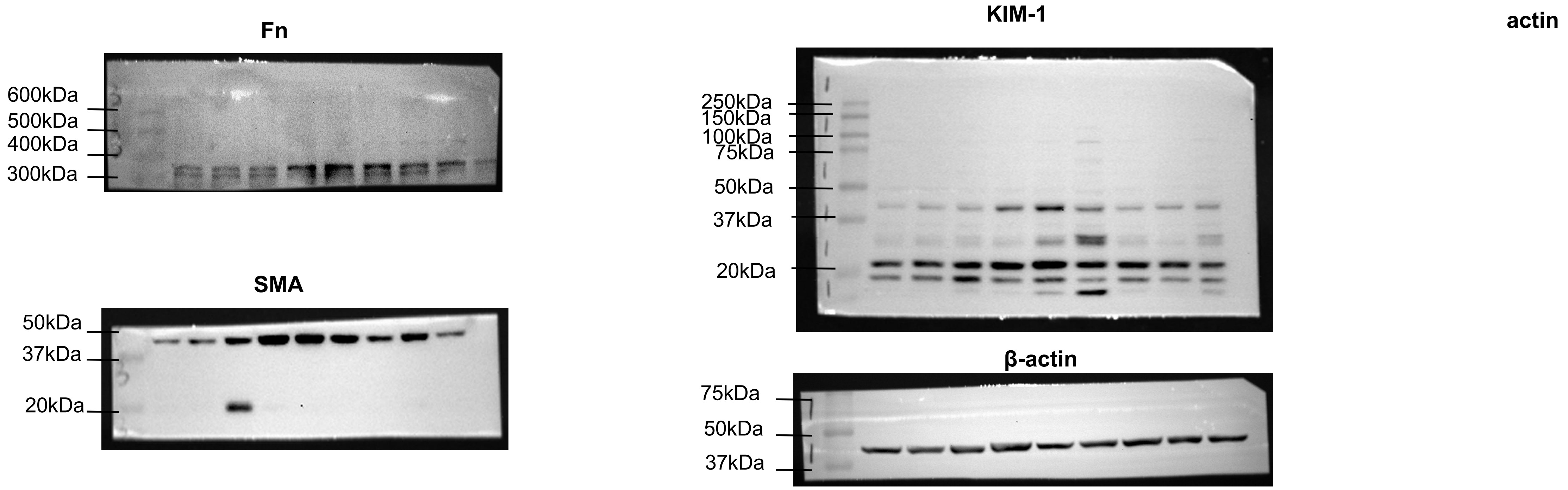

Figure 4B

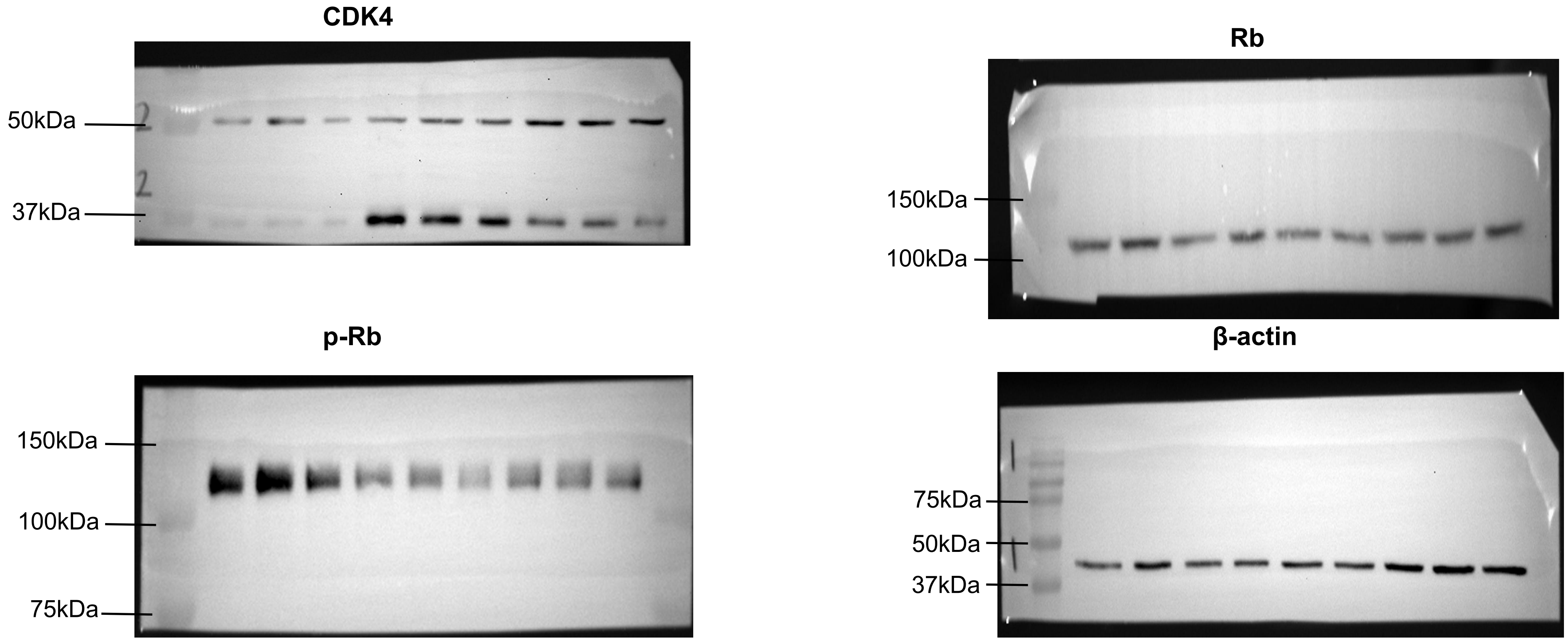
CDK4
50kDa
37kDa
Rb
150kDa
100kDa
p-Rb
150kDa
100kDa
75kDa
β-actin
75kDa
50kDa
37kDa

Figure 7A

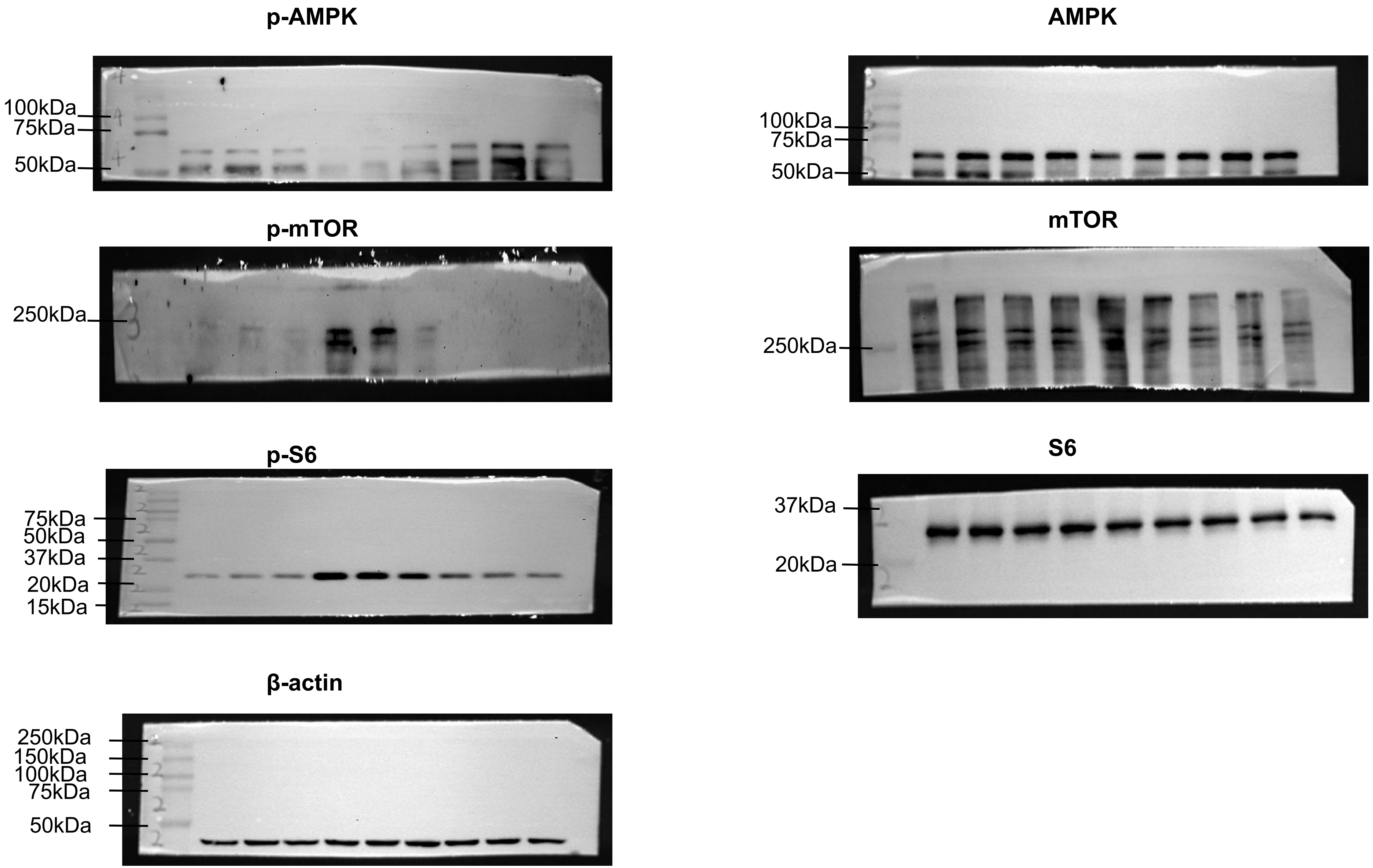
p-AMPK
100kDa
75kDa
50kDa
AMPK
100kDa
75kDa
50kDa
p-mTOR
250kDa
mTOR
250kDa
p-S6
75kDa
50kDa
37kDa
20kDa
15kDa
S6
37kDa
20kDa
β-actin
250kDa
150kDa
100kDa
75kDa
50kDa

Figure 7B

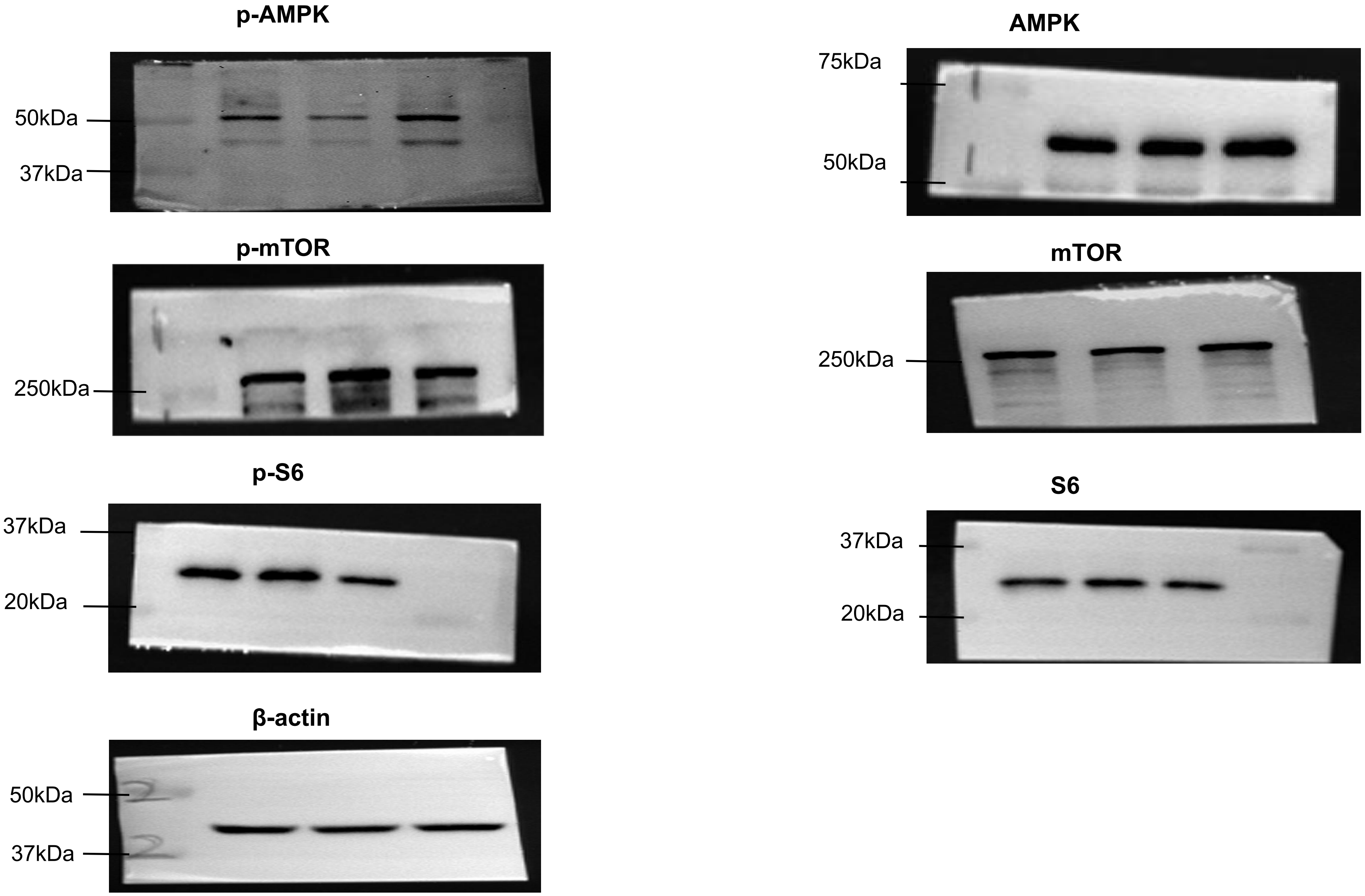
p-AMPK
50kDa
37kDa
AMPK
75kDa
50kDa
p-mTOR
250kDa
mTOR
250kDa
p-S6
37kDa
20kDa
S6
37kDa
20kDa
β-actin
50kDa
37kDa

Figure 8B

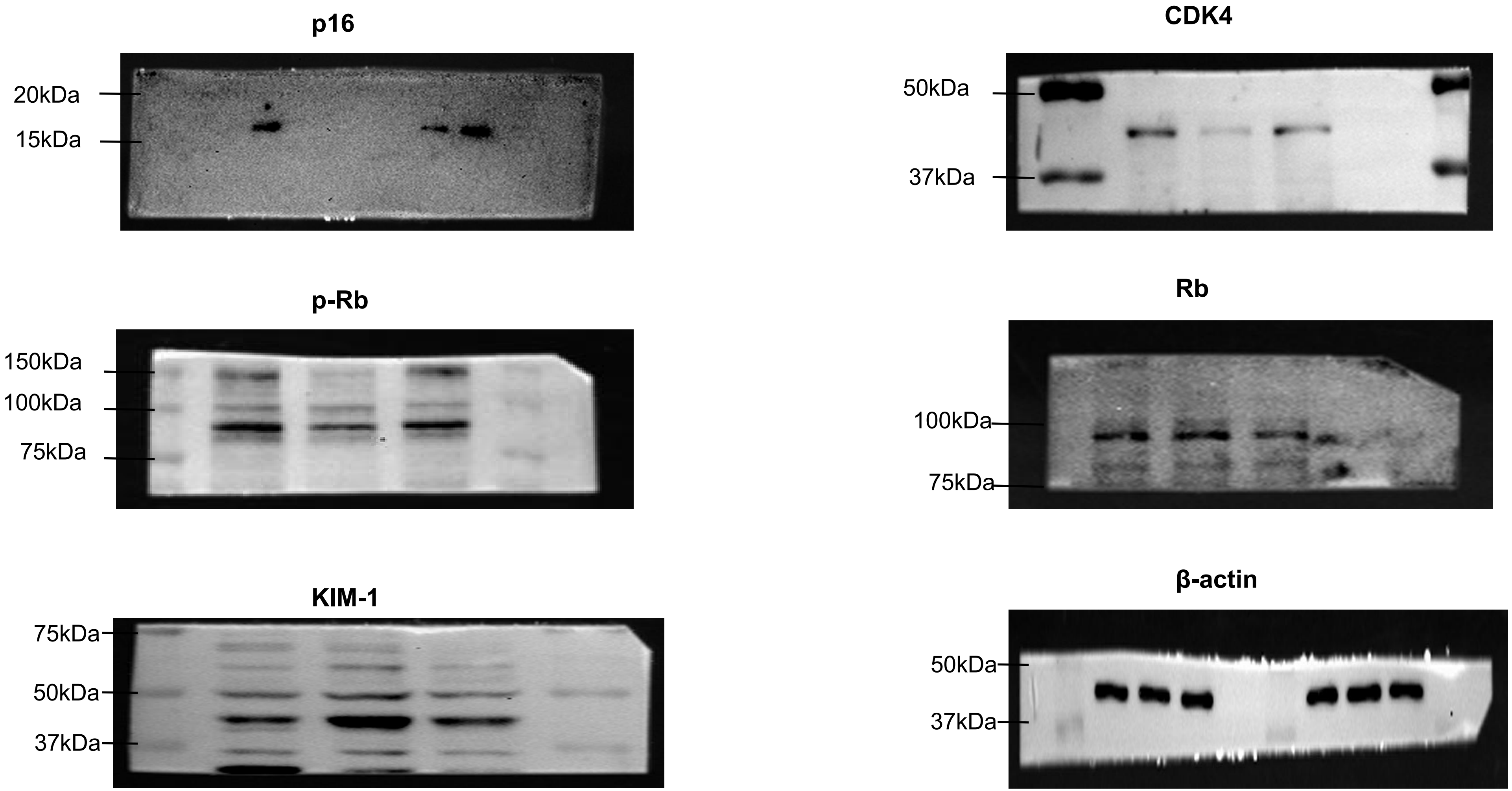

p16
20kDa
15kDa
CDK4
50kDa
37kDa
p-Rb
150kDa
100kDa
75kDa
Rb
100kDa
75kDa
KIM-1
75kDa
50kDa
37kDa
β-actin
50kDa
37kDa

Figure 8F

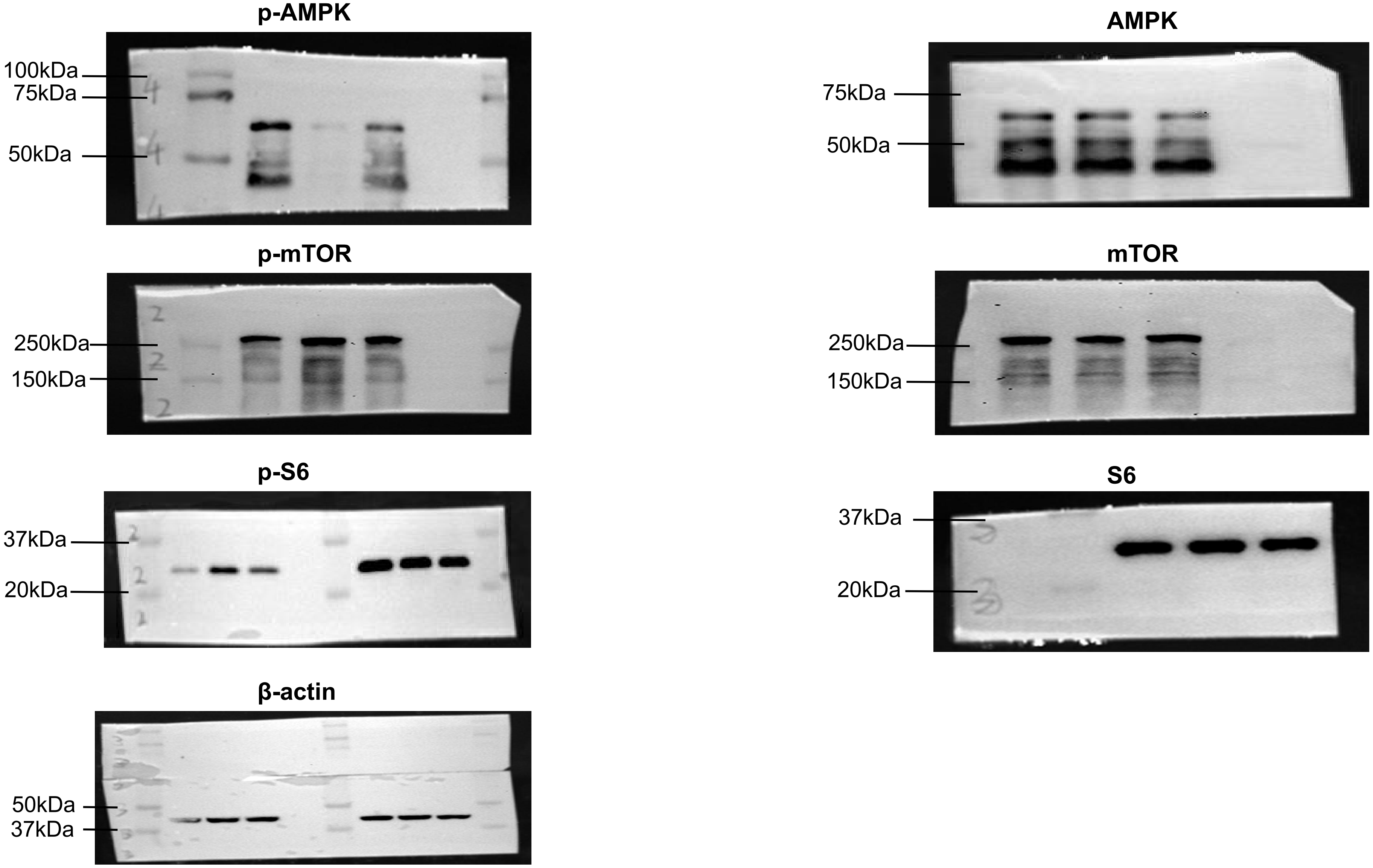
p-AMPK
100kDa
75kDa
50kDa
AMPK
75kDa
50kDa
p-mTOR
250kDa
150kDa
mTOR
250kDa
150kDa
p-S6
37kDa
20kDa
S6
37kDa
20kDa
β-actin
50kDa
37kDa
